# Quantifying intrinsic and extrinsic control of single-cell fates in cancer and stem/progenitor cell pedigrees with competing risks analysis

**DOI:** 10.1038/srep27100

**Published:** 2016-06-01

**Authors:** J. A. Cornwell, R. M. Hallett, S. Auf der Mauer, A. Motazedian, T. Schroeder, J. S. Draper, R. P. Harvey, R. E. Nordon

**Affiliations:** 1 Graduate School of Biomedical Engineering, University of New South Wales, Sydney, NSW 2052, Australia; 2 Developmental and Stem Cell Biology Division, Victor Chang Cardiac Research Institute, Sydney, NSW 2010, Australia; 3 Australian Research Council Special Research Initiative in Stem Cell Science – Stem Cells, Australia; 4 Department of Biochemistry and Biomedical Sciences, McMaster University, Hamilton, ON L8N 3Z5, Canada; 5 McMaster Stem Cell and Cancer Research Institute, Michael G DeGroote School of Medicine, Hamilton, Ontario, Canada; 6 Murdoch Children’s Research Institute, The Royal Children’s Hospital, Parkville, Victoria 3052, Australia; 7 Cell Systems Dynamics, Department of Biosystems Science and Engineering, ETH Zurich, Basel, Switzerland; 8 Department of Pathology and Molecular Medicine, McMaster University, Hamilton, ON L8N 3Z5, Canada; 9 St. Vincent’s Clinical School, Faculty of Medicine, University of New South Wales, Sydney, NSW 2052, Australia; 10 School of Biotechnology and Biomolecular Sciences, University of New South Wales, Sydney, NSW 2052, Australia

## Abstract

The molecular control of cell fate and behaviour is a central theme in biology. Inherent heterogeneity within cell populations requires that control of cell fate is studied at the single-cell level. Time-lapse imaging and single-cell tracking are powerful technologies for acquiring cell lifetime data, allowing quantification of how cell-intrinsic and extrinsic factors control single-cell fates over time. However, cell lifetime data contain complex features. Competing cell fates, censoring, and the possible inter-dependence of competing fates, currently present challenges to modelling cell lifetime data. Thus far such features are largely ignored, resulting in loss of data and introducing a source of bias. Here we show that competing risks and concordance statistics, previously applied to clinical data and the study of genetic influences on life events in twins, respectively, can be used to quantify intrinsic and extrinsic control of single-cell fates. Using these statistics we demonstrate that 1) breast cancer cell fate after chemotherapy is dependent on p53 genotype; 2) granulocyte macrophage progenitors and their differentiated progeny have concordant fates; and 3) cytokines promote self-renewal of cardiac mesenchymal stem cells by symmetric divisions. Therefore, competing risks and concordance statistics provide a robust and unbiased approach for evaluating hypotheses at the single-cell level.

Studying intrinsic and extrinsic control of cell fate and behaviour is necessary to understand stem, progenitor, and cancer cell biology. However, variation in gene and protein expression leading to cellular heterogeneity requires biologists to study cellular systems at single cell resolution^1^,^2^. Time-lapse imaging and cell tracking are invaluable tools that enable cell fate outcomes to be recorded for individual cells^3^. Combined with fluorescent protein reporters, cell tracking provides insight into how a cell’s molecular state interacts with extrinsic stimuli to determine its fate. These technologies have been vital in answering fundamental questions in cell biology^4^,^5^,^6^.

Cell tracking generates cell lifetime data – which contain a record of cell fate and time to fate outcome – as well as kinship (familial relationships), which are visualised as single cell pedigrees (Fig. 1a; see also Supplementary Fig. S1). Figure 1b depicts cell lifetime data in table format for the example pedigree shown in Fig. 1a.

Lifetime data have been used to model cell fate competition^7^, the influence of heritable factors^8^, and cell cycle kinetics^9^. In such models, cell fate is described in probabilistic terms because fate outcomes are not predictable and appear stochastic in heterogeneous populations^10^. Importantly, the probability of a cell adopting a particular fate is determined by its intrinsic state as well as extrinsic factors. Therefore, models of cell growth dynamics must include the influence of cell-intrinsic and extrinsic factors, as well as their interactions, on probabilistic estimates of single-cell fate outcomes.

To accurately quantify probabilistic cell fate outcomes requires one to consider a number of features of cell lifetime data: 1) cell fates may be unobserved (right censored); 2) cell fates may be in competition; 3) cell fates can be concordant in related cells; and 4) distinct cell fates may be intrinsically coupled^11^,^12^.

*Right censoring* refers to when a cell’s final fate is not observed - this occurs when a cell’s trajectory becomes ambiguous, if a cell exits the field-of-view, or when a cell’s fate was not recorded before the end of the observation period. Censored lifetimes are often discarded, although do contain information on whether a cell’s fate was realised before it was censored (see Fig. 1c). *Competing cell fates* are mutually exclusive fates, such that if one fate occurs the other is by necessity censored (e.g. division vs death). Such competition is identical to that defined for mutually exclusive endpoints for patients in clinical trials (e.g. patient death from cancer or from treatment condition). *Concordance* indicates temporal symmetry in the fate of kin, useful to determine if cell fate determinants are inherited. Cell fate outcomes may be determined by *independent* or intrinsically coupled (*dependent*) processes, such that commitment to one outcome may influence the probability of an alternative outcome.

Studies to date have largely excluded cell lifetimes that are right censored or ignored censoring due to competition, leading to significant loss of data and the introduction of bias to the quantification of cell fate. For example, if one restricts analyses to subsets of data where a final fate is observed (e.g., to calculate mean cell cycle times), results will be biased towards shorter lifetimes because longer lifetimes that have been right-censored are under sampled (Fig. 1c). This problem is overcome by the Kaplan-Meier (KM) estimator^13^,^14^ which includes right-censored cell lifetimes to estimate cell fate probabilities. However, the utility of the KM is limited to instances when there is a single fate outcome (e.g., all cells divide or all cells die), and will overestimate the probability of individual outcomes if there are competing fates^15^,^16^. For example, if one applies KM analysis to quantify cell division then cell death outcomes would be considered right-censored (i.e. a cell which has not yet divided). Furthermore, the exclusion of right-censored data affects contemporary methods for analysing concordance in the fate of kin. For example, Pearson’s correlation coefficient only includes cell pairs with known fate outcomes and, therefore, it is biased towards symmetric fate outcomes (Fig. 1c and Table 1). Thus, exclusion of cellular lifetimes may have unintended consequences when quantifying the effect of intrinsic or extrinsic factors on cell fate outcomes and concordance in cell fate. To prevent such selection bias, a method that includes *all* cell lifetimes to quantify cell fate is necessary.

Competing risks (CR) analysis is a method routinely applied to clinical patient data which contain similar features to cell lifetime data^15^. Patients in a clinical trial have competing fates (e.g., death from cancer versus from the cancer treatment) which may be right censored (e.g., patient leaves trial or trial ends)^16^. CR analysis enables one to quantify the probability of each competing fate over time, as well as how the probability of a specific fate outcome is influenced by extrinsic or intrinsic factors^15^. This is achieved by the development of CR regression models that estimate a cumulative incidence function (CIF) for each competing risk (see Supplementary Text 1).

As mentioned above, cell fate outcomes are said to be in competition because only one fate is observed for each cell (e.g. division vs death). Thus, in statistical modelling, observed CIFs are derived from pre-competition probability distributions that have been shaped by censoring^17^. In such a model the first fate to occur determines the observed cell fate while the alternate outcome is censored. However, one cannot determine the dependence structure of the pre-competition multivariate distribution from observed CIFs^18^,^19^. A Monte Carlo simulation illustrating this principle is shown in Fig. 2. The observed division (or death) times were identically distributed, however their bivariate pre-competition distributions had different correlation coefficients (Fig. 2a, r = 0.39, Fig. 2b, r = 0.96). CR analysis accurately estimates the cumulative incidence of the observed competing fates (Fig. 2c) from the observed data, with no dependence on the correlation coefficient. However, KM overestimates the cumulative incidence function for competing fates and is dependent on the correlation coefficient (Fig. 2d). KM was applied by making the erroneous assumptions that a) division and death are independent and b) division right censors death and vice versa. Therefore, unlike other statistics, CR analysis addresses the potential that cell fate outcomes may be coupled. Furthermore, in contrast to the KM method CR analysis provides a more accurate estimate of individual fate outcomes if they are in competition.

CR regression (CRR) estimates how a covariate affects the probability of competing outcomes. Scheike and Zhang developed flexible methods to construct CRR models for clinical patient data^18^. Analogously, we develop CRR models to study cell lifetime data (Supplementary Text 1). Scheike and Sun applied concordance probability statistics to model heritable influences on lifetime events in monozygotic and dizygotic twins^19^. For example, they show that menopause was concordant in monozygotic twins. Analogously, one could apply CR concordance analysis to quantify concordant fates amongst cellular kin (siblings; mother-daughter; 1^st^ cousins; 2^nd^ cousins) to determine if latent, heritable factors control cell fate (Supplementary Text 1d).

Here we apply CR analysis to cell lifetime data including 1) construction of non-parametric and semi-parametric regression models that describe the *effect* of intrinsic and extrinsic factors (covariates) on cell fates; 2) statistical tests to determine whether covariate effects are *significant*; and 3) kinship clustering and concordance analysis to quantify *inheritance* of cell fate. Detailed descriptions of these methods are provided in Supplementary Text 1. This study also compares CR statistics to contemporary statistical tools applied to cell lifetime data, to demonstrate that CR analysis is an improved approach to unbiased testing of biological hypotheses at the single-cell level (Table 1).

We draw upon two existing datasets and generate one novel dataset to demonstrate the utility of CR analysis for testing specific biological hypotheses. In our first analysis we apply CRR models to confirm that wild-type (WT) p53 protects breast cancer (BC) cells against chemotherapy^20^. Clinical efficacy of chemotherapeutic treatments relies on inducing death rather than halting division. The response of a BC cell to chemotherapy depends upon its p53 status, an important consideration for clinicians^21^. Hallett *et al*. applied time-lapse imaging to compare WT p53 and mutant p53 (MUT) BC cell responses to doxorubicin (Dox) or Nutlin3A (Nut)^20^. Nut inhibits the interaction between p53 and Mdm2, an E3-ubiquitin ligase, stabilizing p53 and preventing S-phase entry. Dox is genotoxic and thus induces DNA damage. Thus, in this analysis we apply contemporary statistical tools and CR analysis to the Hallet *et al*. dataset to study the effect of chemotherapeutics and p53 status on division and death in BC cells.

In our second analysis we introduce CR concordance statistics by studying the association between division and differentiation times in cellular kin derived from granulocyte/macrophage progenitors (GMP)^22^. Vertical transmission of molecular determinants of cell fate (RNA and protein expression, epigenetic modifications, etc.) varies over different time scales and spans multiple generations^23^. Inherited traits in related cells are reflected in their concordant fates. Concordance in fate for 1^st^ or 2^nd^ cousins infers inheritance from a common grandparent, or great-grandparent, which have been transmitted over two or three generations (Supplementary Fig. S1). Here, we contrast existing methods for quantifying association in cell fate with CR concordance statistics using data that describe GMP proliferation and differentiation in response to hematopoietic cytokines^22^.

In our third and principal analysis, we utilize factorial design experiments and CRR models to quantify the effects of extrinsic cytokines on division, death, and self-renewal of cardiac colony-forming units – fibroblast (cCFU-F). cCFU-F are a subset of SCA1^+^/PDGFRα^+^/CD31^−^ cardiac interstitial cells in adult mice and have properties of stem cells^24^. cCFU-F are quiescent *in vivo;* however when plated they proliferate and their *in vitro* descendants can differentiate into multiple mesodermal lineages. cCFU-F share a similar gene expression signature to bone marrow-mesenchymal stem cells (BM-MSC) as shown by comprehensive immunophenotype and transcriptome analyses^25^. By employing single cell transcriptome analysis, Noseda *et al*. recently confirmed that within the adult murine myocardium, PDGFRα expression demarcates clonogenic cells within the SCA1^+^ interstitial population^24^,^26^. Here, we apply CRR to investigate how cytokines stimulate self-renewal of PDGFRα^+^ cCFU-F, and apply CR concordance analysis to show for the first time that PDGFRα^+^ cCFU-F self-renew by symmetric cell divisions.

Thus we aim to provide a statistical toolkit for biologists to test hypothesis at the single-cell level without bias. The CR methods described within will improve the accuracy of population-level modelling of cell growth dynamics from single-cell observations, by providing greater insight into the mechanistic control of single cell fate decisions.

## Results

### p53 mediates competing cell fate outcomes in BC cells in response to chemotherapy

Chemotherapy treatment modulated the relative frequency of division, death, and right-censored outcomes (Fig. 3a). Analysis of cell cycle times (Fig. 3b) showed that the mean cell cycle duration was significantly shorter in WT cells than MUT cells in both control (17.9 ± 8.6 hrs in WT vs 25.6 ± 12.6 hrs in MUT, p < 0.001, ±SD) and Nut treated groups (4.1 ± 2.5 hrs in WT vs 32.56 ± 16.7 hrs in MUT, p < 0.0001, ±SD). This result revealed the effect of genotype and chemotherapy on cell cycle times. However, a large proportion of cell lifetimes (up to 87.3%) were not included in the analysis because they were right censored (Fig. 3a), introducing a strong source of bias.

To quantify how intrinsic (p53 status) and extrinsic (chemotherapy) factors (covariates) affected division and death probabilities we constructed a non-parametric CRR linear regression model (see Supplementary Text 2). This model, presented using Wilkinson-Rogers notation^27^, is shown in equation (1)


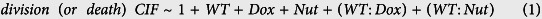


In this model the 1 represents the baseline CIF of untreated MUT cells (Fig. 3c,d, black dashed lines), and a colon represents the interaction between two covariates (e.g. WT:Dox). To generate CIFs for individual factors, or combination of factors, their effects were added to the baseline CIF (see Supplementary Text 2). Qualitative analysis of empirical CIFs revealed that Nut treated WT cells (Fig. 3c, red dashed lines) had reduced division probability relative to untreated WT cells (green dashed lines), Nut treated MUT cells (light pink dashed lines), and untreated MUT cells (black dashed lines). In contrast, Nut treatment did not change the death probability of WT cells (Fig. 3d, red dashed lines) relative to untreated WT cells (green dashed lines), Nut treated MUT cells (light pink dashed lines) or untreated MUT cells (black dashed lines).

To quantify the effect of each factor on division and death semi-parametric models were developed using the method described in Supplementary Text 1c. Semi-parametric models were used to estimate coefficients for each variable in the model if the variable was shown to have constant effects over time (Supplementary Text 2a, and Table S1–S3). The estimated coefficients quantified the magnitude of the effect that each variable had on the outcome of interest, i.e. division or death. The CRR models for division and death are shown in equation (2) and equation (3), respectively.


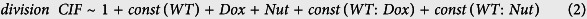



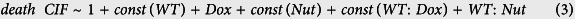


In these models *const*() indicates parametric terms that had constant coefficients. These models revealed that Nut treatment of WT cells reduced division probability by a factor of −1.15 ± 0.22 (Fig. 3c solid green vs solid red lines, p < 0.001, ±SE). Nut also reduced the division probability for MUT cells, though the effect was much smaller (Fig. 3c solid black vs solid light pink lines, p < 0.01). The probability of death was not significantly affected by Nut (Fig. 3d and Supplementary Table S3).

Figure 3c shows that Dox reduced division probability in both MUT (solid black vs solid pink lines, p < 0.001) and WT cells (solid green vs solid blue lines, −0.58 ± 0.20, p < 0.005). In contrast, Fig. 3d shows that Dox increased death probability in MUT cells (solid black vs solid pink lines, p < 0.001) but not WT cells. Notably, WT cells had a proliferative advantage over MUT cells as they had a higher division probability in controls (Fig. 3c solid green vs solid black lines 0.63 ± 0.20, p < 0.005).

These analyses demonstrate how CR statistics allow cell fate outcomes to be quantified by including every cell’s lifetime, rather than a subset of observed outcomes. This is an important contribution because on average across all groups 41.7% of BC cell fate outcomes were unobserved (Fig. 3a). Furthermore, CR analysis doesn’t assume that CR are independent^28^,^29^; in contrast to other parametric models that consider death and division to be independent outcomes^17^. These results also confirmed the dependence of p53 mediated death and division pathways in BC cells, further highlighting that consideration of such dependency will improve the efficacy of chemotherapeutic treatments.

### CR concordance analysis quantifies the influence of latent, heritable determinants on cell fate in GMP kinship clusters

**Figure 1:**
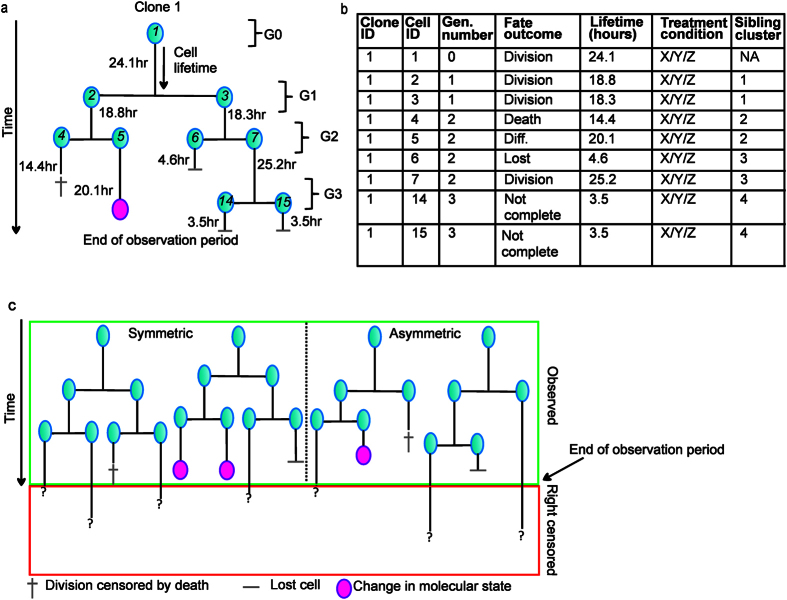
Single-cell tracking generates cell lifetime data that are visualized as single-cell pedigrees. (**a**) Stylised cartoon of a single cell pedigree showing the fate and time of fate for each cell (right censored, lost, division, death, differentiation, etc.), kinship relationships, and generation numbers. Measurements of a cell’s internal molecular state, morphological appearance, as well as other lifetime events such as cell-cell contact may be recorded within a single cell pedigree. Establishment of kinship relationships within a single cell pedigree provides unique access to study the influence of inheritance on cell fate outcomes. (**b**) Cell lifetime data from the pedigree shown in (**a**), depicted in table format. Note: only sibling cell clusters are shown in the table. (**c**) Example of heterogeneous fate outcomes and right censoring that is inherent within single cell pedigrees. The green and red boxes demarcate observed and right censored fates. Lost cells are also considered to be right censored because their final fate is not observed. The two pedigrees on the left of the grey dotted line exemplify symmetric fate outcomes in siblings, i.e. the pair of daughter cells produced by the first division in each pedigree divide at the same time. The two pedigrees on the right of the grey dotted line exemplify asymmetric fate outcomes in siblings, i.e. the pair of daughter cells produced by the first division in each pedigree have discordant fates as a result of right censoring and competing fate outcomes. Importantly, if censored lifetimes and discordant fates are discarded from cell lifetime analysis (i.e. only pedigrees on the left of the grey dotted line would be included) then conclusions are biased towards symmetric fates and shorter cell lifetimes.

Correlation analyses - Pearson’s correlation coefficient (PCC) and intraclass correlation (ICC) – measure temporal association in fate outcomes in related cells from paired observations^8^,^9^,^17^. Permutation tests compare fate outcomes of kin to randomly sampled cell pairs to test for significant associations within kinship clusters^12^,^30^. Such statistics biasedly select symmetric fate outcomes because right-censored and discordant fates are excluded (Table 1, Fig. 1c). Yule’s Q^17^ and the binomial test^12^ measure concordance using the relative frequency of cell fate outcomes, but exclude right-censored fates (Table 1, Fig. 1c). Thus, such tests limit the detection of asymmetric outcomes and do not allow one to study temporal association in fate, making it difficult to attribute concordance in cell fate to heritable fate determinants.

We propose CR concordance probability and the cross-odds ratio (COR) as unbiased methods to study association in cell fate. These methods include right-censored lifetimes, CR, and a clustering variable that denotes kinship relationships (Supplementary Text 1d and Fig. S1). The COR measures how timely occurrence of one event affects the odds of another^19^. Here the COR was utilized to measure how the occurrence of an event for one cell (e.g., time to division or differentiation) affected the odds of the same event occurring in its kin. The COR is a simple measure; a COR > 1 indicates concordant fates and a COR < 1 indicates discordant fates. Here, the null hypothesis is that cell fates are independent (COR = 1). Additionally, we demonstrate that CR concordance analysis uniquely allows one to visualise how association in fate varies over time (Supplementary Text 1d).

We apply our analysis to GMPs that were isolated from LysM::GFP mice, differentiated using either GCSF or MCSF, and tracked up to 7 generations^22^. LysM::GFP reported lysozyme expression and was used to time onset of uni-lineage commitment for both granulocyte and macrophage fate^6^. Firstly, to understand the influence of growth factors (GF = 0 *or* 1, GCSF *or* MSCF, respectively) and differentiation (GFP = 0 *or* 1) on division and death probabilities we constructed a non-parametric CRR model, shown in equation (4).


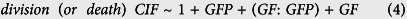


This model showed that GCSF and MCSF had indistinguishable effects on division and death probabilities (Fig. 4a,b, black line), suggesting these extrinsic factors have the same effect on division and death outcomes in GMPs. In contrast, after differentiation into macrophage and granulocyte progeny both division and death probabilities were significantly altered (Fig. 4a,b). These results suggested that the growth factor specific effects on division and death outcomes are latent until lineage commitment (GFP onset).

Concordance in cell fate was then investigated by constructing CRR models that included a clustering variable to identify cell kinship (Table 2 and Supplementary Text 3). The COR shown in Table 2 reveal that time to division was highly concordant within all kinship clusters. The degree of concordance was inversely related to the number of divisions between cells in the cluster and their common ancestor (i.e. siblings >1^st^ cousins >2^nd^ cousins). This trend was visualised by plotting the conditional and unconditional probabilities (Fig. 4c,d). COR for undifferentiated cells (GFP^−^) were 20.9 ± 0.61 for siblings; 3.7 ± 0.9 for 1^st^ cousins; 3 ± 0.63 for 2^nd^ cousins. We observed weaker concordance in differentiated cells (GFP^+^)−3.71 ± 0.66 for siblings; 2.1 ± 0.2 for 1^st^ cousins; 1.38 ± 0.12 for 2^nd^ cousins. Pearson’s correlation analysis revealed a similar pattern of correlated time to fate (Supplementary Fig. S2 and Fig. S3).

Concordance was weaker between mothers and daughters than for cells within the same generation, indicating greater variation in intergenerational times than intragenerational times; COR were 0.53 ± 0.16 and −0.19 ± 0.07 for GFP^−^ and GFP^+^ cells, respectively (Table 2 and Fig. 4c,d, green lines). Negative COR for GFP^−^ mother-daughter clusters indicated discordant fates, attributed to longer cell cycle times in differentiated cells that lead to greater discordance in fate outcomes (Supplementary Fig. S2 and Fig. S3).

Onset of GFP fluorescence and differentiation occurred in generations 0–6 over a 7 day period, but was synchronized in cells with a common ancestor (Fig. 4e,f). COR for onset of GFP expression, taken from the time the stimulus was received by ancestors, were 1.3 ± 0.35, 3.4 ± 0.23 and 2.9 ± 0.16 for sisters, 1^st^ cousins and 2^nd^ cousins, respectively (Table 2). Correlation analysis corroborated these findings: r = 0.95, 0.91, and 0.88 for siblings, 1^st^ cousins, and 2^nd^ cousins, respectively (Supplementary Fig. S4), though on average only half of all pairs were analysed because right censored and discordant fates were excluded (Fig. 4g,h).

Excluding censored cell fates resulted in significant selection bias in all kinship clusters (Fig. 4g). For example, when analysing time to division for GFP- GMPs Yule’s Q test quantified concordance in cell fate, while ICC and PCC quantified correlation in time to fate (Supplementary Table S4). However, these tests only included limited subsets of cell lifetime data (Fig. 4h and Supplementary Table S4). CR concordance analysis included all cell observations and therefore is an unbiased method for quantifying the influence of latent, heritable determinants on cell fate.

### bFGF, PDGF, and TGFβ1 stimulate cardiac MSC self-renewal by symmetric PDGFRα^+^ divisions

Proliferation and colony-forming assays are routinely used to measure self-renewal of CFU-F from BM and other tissues^31^. These assays rely on population snapshots and therefore obscure the underlying detail of dynamic single-cell behaviour^3^,^32^. Studying single cell behaviour is critical since a cell’s response to one cytokine may depend on the presence of other cytokines via interacting intracellular signalling pathways^33^. Here, we study the effect of cytokine stimulation on cCFU-F division, death, and self-renewal.

cCFU-F were isolated from 8–12 week old *Pdgfra*^*GFP*/+^ knockin mouse hearts, which express a nuclear version of GFP under *Pdgfra* cis-regulatory control. cCFU-F were expanded in serum through 3 passages and then exposed to basic fibroblast growth factor (bFGF), platelet-derived growth factor (PDGF), and transforming growth factor-beta 1 (TGFβ1) in serum free medium (SFM). These growth factors were selected as their ability to stimulate MSC proliferation and self-renewal is well characterised^34^. Cells were observed by time-lapse microscopy and single cell tracking for 96 hours. We observed GFP^−^ and GFP^+^ cells that had fibroblast morphology, as well as larger, myofibroblast-like cells that were GFP^−^ (Fig. 5a). Interactions between intrinsic *Pdgfra*-GFP expression and cytokine treatment were resolved using a two-level (present or absent), full-factorial (all combinations) experimental design (Supplementary Text 4 and Table S5). We applied CRR models to quantify cytokine effects on division and death (Supplementary Text 5a), as well as the effect of interactions between cytokine stimulation and *Pdgfra*-GFP expression (Supplementary Text 5b). CRR models and concordance analysis were then used to analyse symmetric and asymmetric self-renewal of cCFU-F (Supplementary Text 5c–e). Results derived from CRR models were also validated by population-based proliferation and CFU-F assays.

First, we constructed a CRR model to quantify single factor, as well as two factor (bFGF:PDGF; PDGF:TGFβ1; bFGF:TGFβ1) and three factor (bFGF:PDGF:TGFβ1) interactions on division probability for generation 0 cells (see equation (5)).


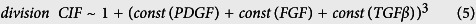


The model showed that the division probability was greater with all three factors, compared to bFGF, PDGF or TGFβ1 alone (Fig. 5b). There was synergy between these cytokines because bFGF:PDGF:TGFβ1 had a larger effect (2.55 ± 0.76) than the combined effects of single factors (TGFβ1; 0.249 ± 0.11; PDGF: 0.4 ± 0.12; and bFGF: 0.49 ± 0.15) or no factors (Fig. 5b, Supplementary Fig. S5 and Text 5a). Empiric CIFs followed the same trend, demonstrating goodness-of-fit of the parametric model (Supplementary Fig. S5 and Text 5a). Similar observations were made for cells whose birth was observed (generation >0, Supplementary Fig. S5). Population-based proliferation and CFU-F assays validated the results derived from our model (Supplementary Text 4 and Fig. S6). Without factors cells did not proliferate, while bFGF alone had the greatest (p < 0.001), and TGFβ1 alone had the smallest effect on division probability (p = 0.003), respectively. Effects on survival were screened; only TGFβ1 increased death probability (Supplementary Fig. S5 and Text 5a).

To investigate renewal of the cCFU-F phenotype (defined by SCA1^+^/PDGFRα^+^/CD31^−^ expression) we showed that cCFU-F cultures did not lose the stem cell marker SCA-1, nor gain the endothelial marker CD31 (Supplementary Table S22). However, single cell *Pdgfra*-GFP expression varied considerably and was related to cytokine treatment (Fig. 5c and Supplementary Fig. S7). Thus, PDGFRα expression could be used to identify renewal of the cCFU-F phenotype, as reported by Chong *et al*.^24^ and confirmed by Noseda *et al*.^26^.

We developed CRR models that included a variable for GFP intensity in order to study the interaction effects of cytokines on *Pdgfra* expression (Supplementary Text 5b). These models showed that of the cytokines promoting division, only PDGF had a significant positive interaction with GFP expression (p < 0.001, Supplementary Text 5b). In contrast, GFP expression was not related to death probability. To classify cells as GFP^+^ and GFP^−^ we established a threshold of GFP expression, below which PDGF did not affect division probability (Fig. 5c,d and Supplementary Text 5b). We observed a relatively constant proportion of GFP^+^ cells in each generation (Supplementary Fig. S7), which was hypothesised to be achieved by self-renewal of GFP^+^ cells.

To study renewal of GFP^+^ cells we divided cell fate outcomes into four CR categories: 1. GFP^+^ division; 2. GFP^−^ division; 3. GFP^+^ death; and 4. GFP^−^ death (Fig. 5e and Supplementary Text 5c). The CRR model showed that division probability for GFP^+^ cells treated with PDGF:bFGF (the factors of greatest effect) was >0.5, while division probability of GFP^−^ cells treated with bFGF (knowing PDGF had no effect) was <0.2 (Fig. 5f and Supplementary Text 5c). Figure 5g shows that GFP^+^ cells were derived mostly from GFP^+^ mothers (50.7%) but also GFP^−^ mothers (6.8%), while GFP^−^ cells were derived equally from GFP^+^ and GFP^−^ mothers (20.4% and 22.1%, respectively). This agreed with our CRR model (Fig. 5h and Supplementary Fig. S7). Taken together, these findings confirmed that GFP^+^ cells self-renew (probability > 0.5), while GFP^−^ cells do not renew over multiple generations because their renewal probability is less than 0.5. We also validated functional self-renewal of cCFU-F cultured in bFGF, PDGF and TGFβ1 by demonstrating maintenance of *in vitro* lineage potency (data not shown).

We next investigated if GFP^+^ cells self-renew symmetrically or asymmetrically (Fig. 6a). Firstly, we divided sibling pairs (for all sibling pairs and GFP^+^ pairs) into groups where both siblings divided, both died, fates were discordant, or where at least one sibling’s fate was right censored (Fig. 6b). We observed strong correlation in time to division for all siblings pairs and GFP^+^ pairs (all pairs: ICC = 0.78, and r = 0.71, p < 0.001; GFP^+^ pairs: ICC = 0.89, r  = 0.79, p < 0.001, Fig. 6b,c). Yule’s Q also estimated strong concordance in fate for both groups (all pairs: Q = 0.79, GFP^+^: Q = 0.89, Fig. 6b). Figure 6d shows that the mean difference in cycle times between cell pairs from random permutations was much greater than that of the observed mean difference between sibling pairs (all pairs: 18.37 ± 1.24 hrs vs 8.59 hrs, t = 10.19, p << 0.001, GFP^+^ pairs:16.72 ± 1.34 hrs vs 7.16 hrs, t = 10.23, p << 0.001, see Supplementary Text 6). These different tests all demonstrated strong correlation and concordance in fate of siblings; however they are inherently biased toward symmetric fate outcomes (Table 1). Therefore, we applied CR concordance analysis to quantifying the COR for GFP^+^ sibling cell divisions^19^. This analysis included right censored data as well as temporal information regarding time to fate, to quantify synchronicity in sibling cell divisions (Fig. 6a and Supplementary Text 5e).

We established whether the conditional probability of a GFP^+^ division for one cell was increased given that its sibling was GFP^+^ and had divided. We constructed CRR models for GFP^+^ divisions that included a cluster term to identify sibling pairs (*SiblingClusterID*), shown in equation (6) (see Supplementary Text 5e).


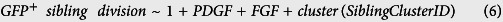


The conditional probability of a GFP^+^ division was significantly higher than the unconditional probability of a GFP^+^ division (Fig. 6e and Supplementary Text 5e), establishing that the probability of a GFP^+^ division for a cell was increased if its sibling was also GFP^+^ and had divided. The COR for GFP^+^ divisions in siblings was 10.7 ± 1.35 (±SE, p < 0.001), demonstrating that cultured cCFU-F self-renew by symmetric divisions using *Pdgfra*-GFP expression as a marker of cell fate.

In conclusion, our analysis demonstrates that bFGF, PDGF and TGFβ-1 are sufficient for cCFU-F self-renewal. Renewal activity resided within the PDGFRα^+^ cell subset, though FGFR and TGFβR are likely co-expressed by PDGFRα^+^ cCFU-F. Concordance analysis showed that these cytokines stimulate self-renewal of PDGFRα^+^ cells by symmetric divisions. CRR and CR concordance analysis thus allowed us to quantify stem cell renewal dynamics at single cell level. Such detailed characterization of cCFU-F will help to shed light on their potential role in remodelling cardiac tissue in response to injury.

## Discussion

**Figure 2:**
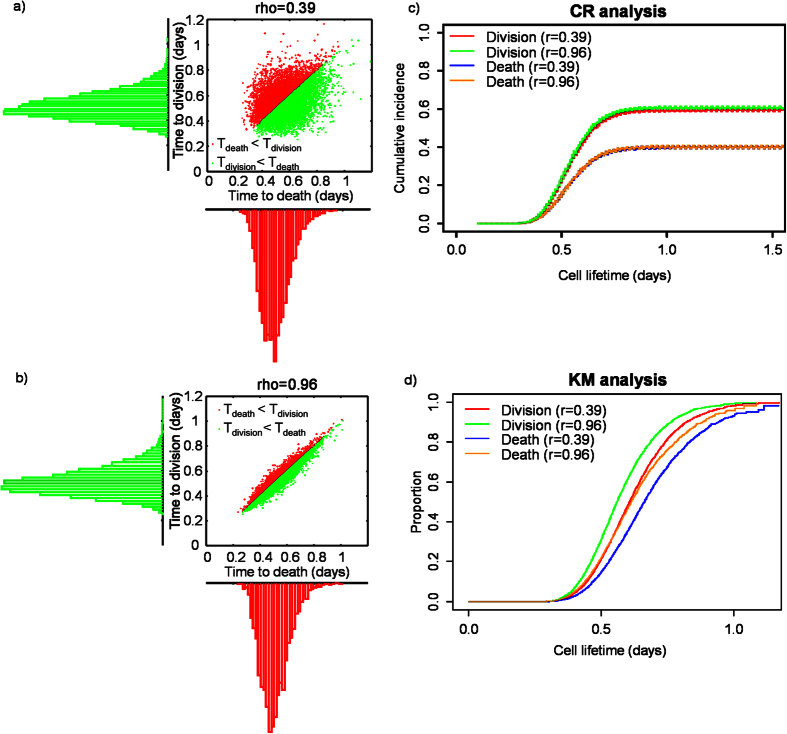
Bivariate plots and histograms demonstrating that pre-competition probability distributions (bivariate plots) cannot be identified from post-competition distributions for division and death (histograms). In (**a,b**) pre-competition distributions show varying degrees of correlation (rho = 0.39 and rho = 0.96, respectively) while observed division and death distributions remain unaffected. (**c**) Competing risks analysis of time to division and death for the distributions shown in (**a,b**). Dotted lines represent 95% confidence intervals. Note that there is no dependence on the correlation coefficient (rho). (**d**) Kaplan-Meier analysis of time to division and death for the distributions shown in (**a,b**). Note that the probability of death and division is over estimated, and there appears to be dependence on the value of the correlation coefficient.

**Figure 3:**
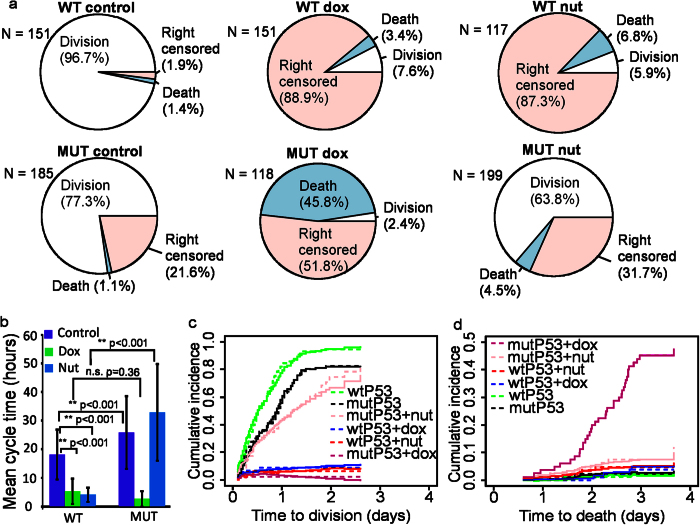
Effect of chemotherapy on division and death of WT (pooled BT474 and MCF7 cell lines) and MUT (pooled MDA-MB231 and T47D cell lines) BC cells treated with Dox or Nut. (**a**) Pie-charts showing the distribution of fate outcomes (division, death, right censored) in control and dox treated WT and MUT BC cells. (**b**) Mean cycle time in control and dox treated WT and MUT BC cells. Right censored lifetimes are not included in calculation of mean cycle time. **Indicates p < 0.001 and n.s. is not significant. (**c**) Non-parametric (dashed lines) and semi-parametric (solid lines) CIF for division probability in WT (green), MUT (black), MUT + Nut (light pink), WT + Dox (blue), WT + Nut (red), and MUT + dox (pink). (**b**) Non-parametric (dashed lines) and semi-parametric (solid lines) CIF for death probability in WT (green), MUT (black), MUT + Nut (light pink), WT + Dox (blue), WT + Nut (red), and MUT + Dox (pink). (**d**) The estimated coefficients for semi-parametric models are shown in Supplementary Table S3. All data analysed are from pooled observations from replicate wells for each condition (N = 853 cells).

**Figure 4:**
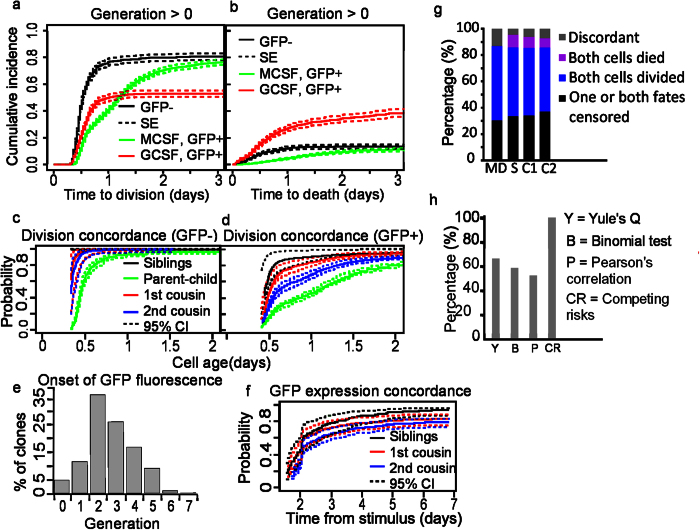
CR regression and concordance analysis of division, death, and differentiation in GMPs treated with hematopoietic cytokines. (**a**) Simulated CIFs for division for GFP- cells (black) treated with MCSF or GCSF, GFP+ cells treated with MCSF (green), and GFP+ cells treated with GCSF (red). Dashed lines show standard error (SE). (**b**) Simulated CIF for death for GFP- cells (black), treated with MCSF or GCSF, GFP+ cells treated with MCSF (green), and GFP+ cells treated with GCSF (red). Dashed lines show SE. (**c**) Division concordance probability for GFP- siblings (black), parent-child (green), 1^st^ cousins (red), and 2^nd^ cousins (blue). Dashed lines are 95% confidence intervals (CI). (**d**) Division concordance probability for GFP+ siblings (black), parent-child (green), 1^st^ cousins (red), and 2^nd^ cousins (blue). (**e**) Histogram showing the number of cells that transition from GFP- to GFP+ in each generation (MCSF and GCSF pooled data). (**f**) Concordance probability for onset of GFP expression after MCSF treatment for siblings (black), 1^st^ cousins (red), and 2^nd^ cousins (blue). Dashed lines are 95% CI. (**g**) Histograms showing the proportion of concordant, discordant, and censored fate outcomes in mother-daughter (MD), sibling (S), 1^st^ cousin (C1), and 2^nd^ cousin (C2) kinship clusters. (**h**) The percentage of cell lifetime data used by statistical tests for quantifying association in cell fate (averaged over all kinship clusters). CRR models and COR are shown in Table 2.

**Figure 5:**
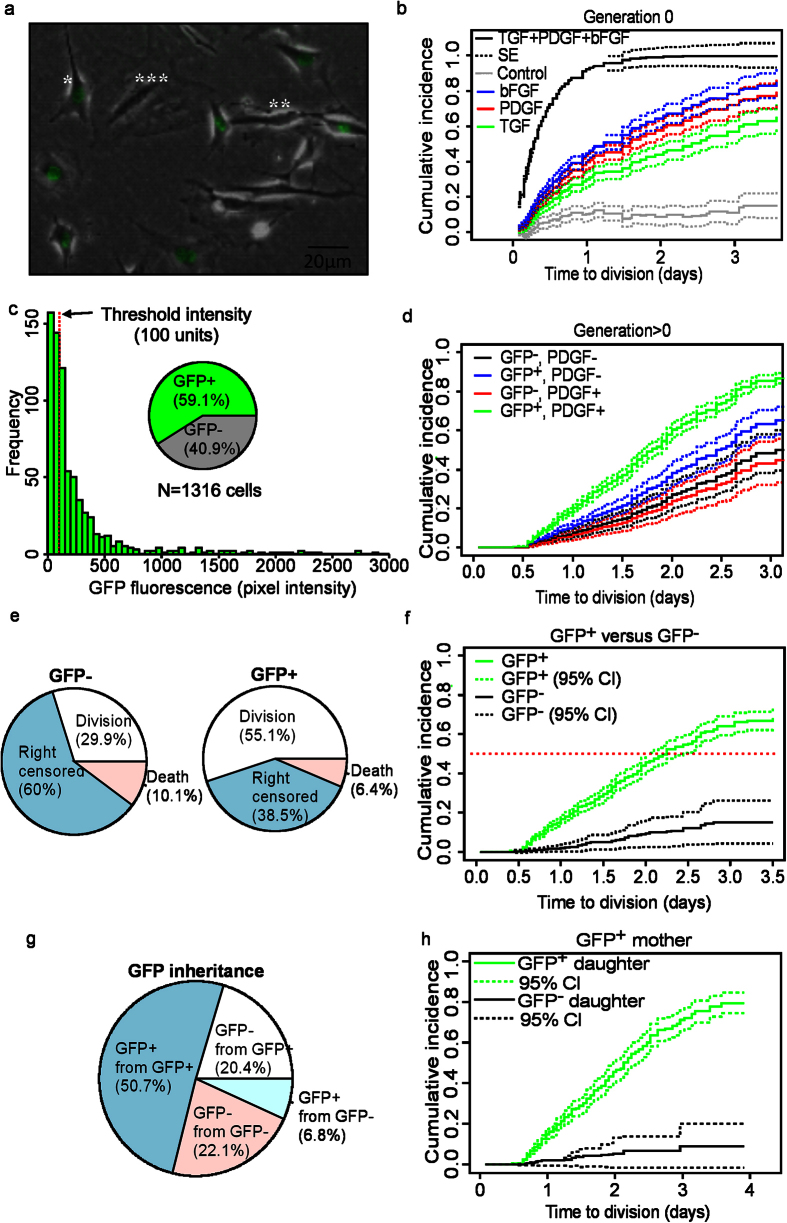
The effect of cytokines and PDGFRα expression on probability of division, death, and self-renewal of cCFU-F. (**a**) Overlaid phase contrast and fluorescence (GFP) image of *Pdgfra*-GFP cCFU-F showing heterogeneity in morphology and GFP expression. *and **indicate GFP^+^ and GFP^−^ spindle-shaped cells, respectively. ***Indicates a GFP^−^ cell with a myofibroblast morphology. (**b**) CIF (solid lines) for division (generation 0) given TGFβ1 (green), bFGF (blue), PDGF(red), all three factors (black), or no factors (grey). Dashed lines show standard errors (SE). (**c**) Histogram showing variation in *Pdgfra*-GFP intensity, and threshold limit used to classify GFP^+^ and GFP^−^ cells. (**d**) Simulated CIF (solid lines) for division (generation > 0) of GFP^+^ cells with PDGF (green), GFP^+^ without PDGF (blue), GFP^−^ cells with PDGF (red), and GFP^−^ cells without PDGF (black). Dashed lines show SE. (**e**) Pie-charts showing the frequency of observed fate outcomes for GFP^+^ and GFP^−^ cells. (**f**) Simulated CIF (solid lines) for GFP^+^ divisions (green) vs GFP^−^ divisions (black). Dashed green and red lines are 95% confidence intervals (CI). (**g**) Pie-chart showing inheritance of GFP. GFP^+^ mothers give rise to a majority of GFP^+^ and a minority of GFP^−^ daughter cells, while GFP^−^ mothers equally give rise to GFP^−^ and GFP^+^ cells. (**h**) Simulated CIF (solid line) showing that GFP^+^ mothers give rise to a majority of GFP^+^ and a minority of GFP^−^ daughters. Dashed lines are 95% CI. All data analysed are from pooled observations from replicate wells for each condition (N = 1316 cells).

**Figure 6:**
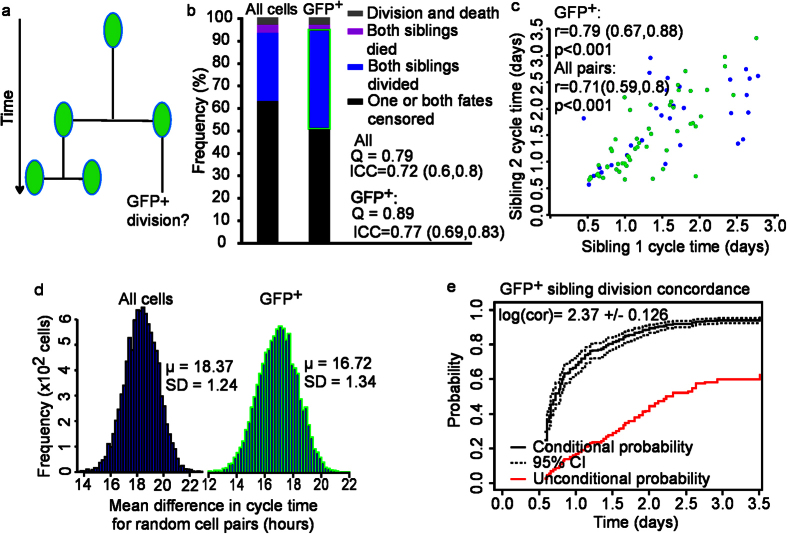
Symmetry in cCFU-F sibling cell fate outcomes. (**a**) Illustration of symmetric GFP^+^ division. (**b**) Relative proportion of discordant, concordant, and right-censored fates for cCFU-F sibling pairs. Q is Yule’s Q estimate of association in fate. ICC is the intraclass correlation coefficient, numbers in brackets are 95% CI. (**c**) PCC in sibling cell divisions, GFP^+^ pairs (green), GFP^−^ pairs (blue). r is Pearson’s correlation coefficient and numbers in brackets are 95% CI. For all cells N = 84 pairs, and for GFP^+^ cells N = 51 pairs. (**d**) Distribution of the mean difference between 10,000 random permutations of cell pairs for all cells (left) and GFP^+^ pairs (right). μ and SD represent the mean and standard deviation of the randomly generated distributions, respectively. The observed mean difference in cell cycle for all siblings was 8.59 hr. The observed mean difference in cell cycle for GFP^+^ pairs was 7.17 hr. In both groups the observed mean difference was significantly shorter than the mean difference between randomly sampled cell pairs. (**e**) Probandwise concordance (black), showing the conditional probability of a GFP^+^ division for sibling 1 given a GFP^+^ division for sibling 2 had occurred (black dashed line, 95% CI). The unconditional probability of a GFP^+^ division (red solid line) is much lower. +/− indicates SD. All data analysed are from pooled observations from replicate wells for each condition (N = 1316 cells).

We have shown that BC cell division and death after treatment with cytotoxic agents are dependent on p53 genotype. Alternatively, one may hypothesise that fate outcomes are determined by independent, autonomous processes that compete within each cell^11^,^17^. While such models have been proposed to describe cell growth dynamics, one is unable to establish dependence between competing fates because in statistical modelling pre-competition distributions cannot be determined from lifetime data alone (Fig. 2)^35^. Importantly, the CR models we presented make no assumption of independence^28^. In the future a CRR model could be enhanced through correlating p53 activities in single BC cells with their fate - since division and death outcomes in BC cells also depend on temporal fluctuations of p53 state^29^.

We describe statistical tools for analysing concordance in cell fate, advancing upon previous methods that do not take into account censorship and competition. Familial concordance in fate has been reported to exist in different cellular systems^9^,^36^. These observations suggest that latent, heritable determinants of fate are transmitted vertically to determine fate and time to fate. Heritable determinants may be signalling components, segregated proteins, and epigenetic or genetic alterations^37^. Dependence of fate in kin and independence of fate in unrelated cells contributes to population heterogeneity, and must be considered for accurate modelling of cell growth dynamics^9^,^11^, e.g. to determine if two heritable factors could explain correlation in time to division and death in lymphocytes^8^. We advance upon previous approaches for quantifying concordance in cell fate by applying CR concordance probability statistics that accommodate right-censored and competing fate outcomes. We quantified concordance in fate for GMP kin, showing that it is stronger for siblings than for 1^st^ and 2^nd^ cousins. An analysis of further removed kin (i.e. 3^rd^ cousins) could determine what degree of familial separation results in loss of concordance. In contrast to reports by Sandler *et al*.^38^, we observed that division times for mother-daughter pairs are also correlated, though more weakly than cells within the same generation, which we attribute to intergenerational cell cycle lengthening; a nascent inherited characteristic that reduces concordance. Macrophage progeny had increased cell cycle lengths and weaker sibling concordance compared to GMPs, similar to that observed during neural stem cell differentiation - where increased cell cycle length increased the frequency of asymmetric divisions in siblings^39^. Weaker concordance may be attributed to divergence of protein synthesis that does not occur during shorter cycle times^40^. Our results support the concept that cell fate outcomes are inheritable, and that the influence of inheritance may be diluted by cell division. In the future, our analysis could be extended by investigating how extrinsic factors influence concordance.

Factorial design and CRR models were applied to the analysis of PDGFRα^+^ renewal within cultures of cCFU-F. CRR showed that bFGF, TGFβ-1, and PDGF synergistically stimulated cCFU-F proliferation and self-renewal. Synergism between these factors has been reported^33^, though not demonstrated in single cells. CRR confirmed that *Pdgfra*-GFP expression marks cells that respond to PDGF signalling. A recent single cell analysis of the SCA1^+^ interstitial fraction of adult murine myocardium confirmed that PDGFRα marks cardiogenic clonogenic cells^24^,^26^. By applying CRR and concordance analysis to cell lifetime data we demonstrated that cultured cCFU-F renew by symmetric PDGFRα^+^ divisions. Given the large number of censored lifetimes and the marked heterogeneity in cCFU-F cultures, this finding would be difficult to prove robustly using the other statistical methods shown in Table 1 because of their selection bias. Our results show that the probability of division for PDGFRα^+^ cCFU-F is increased by stimulation with PDGF. Over the last decades the dogma that the adult mammalian heart is a post-mitotic organ has been overturned, resulting in increased interest in cardiac regeneration. Our results provide insight into what molecular cues could be used to stimulate cCFU-F to actively proliferate and self-renew *in vivo*. In the future such insight may enable development of new therapies that target cCFU-F in order to enhance their response to cardiac injury.

Throughput of single-cell tracking is increasing^3^. Without robust statistical tools it is likely that erroneous conclusions will be drawn through selection bias. CRR offers a comprehensive method to evaluate biological hypotheses at the single cell level using all cell lifetime data. Single cell tracking, transcriptional profiling and CR statistics are essential tools for directly establishing the causative link between molecular pathways and cell fate.

## Materials and Methods

### Isolation and culture of mouse GMPs

All details of isolation and culture of mouse GMPs was described previously^6^.

### Time-lapse imaging and single cell tracking of GMPs

Details of time-lapse imaging and single cell tracking of GMPs has previously been described^6^.

### Cell culture and generation of H2GFOIP reporter lines

Cell culture and generation of H2GFOIP reporter lines has been described previously^20^.

### Live Cell Imaging and Single Cell Tracking of BC Cell Lines

Details for live and high content imaging are described previously^20^.

### Isolation and expansion of cardiac colony forming unit (cCFU)

cCFU were isolated from 8–12 week old *PDGFRa*-GFP transgenic C57/BL6 mice, as previously described^24^. After FACS cells were collected in medium containing 20% FCS. Cells were expanded in 20% serum by plating at 250 cells/cm^2^ in 10 cm dishes coated with gelatin. For live cell imaging experiments, cells at passage 4 were plated at 250 cells/cm^2^ in 24-well live cell imaging plates (Ibidi) and exposed to factorial combinations of cytokines in SFM. See Supplementary Text 4 for details on the design and analysis of factorial design experiments.

### Colony Forming Unit (CFU) Assay

Passage 4 cells were plated at 50 cells/cm^2^ in 35 mm dishes in triplicates. After 8 days cells were stained with crystal violet to visualise colonies, as described previously^24^. Colonies with more than 25 cells and greater than 2 mm in diameter were counted.

### Proliferation

Passage 4 cells were plated at 200 cells/cm^2^ in 35 mm dishes in triplicates. After 6 days in culture (or when cells reached 80% confluence) cells counted. Each replicate was counted twice.

### Live Cell Imaging and Single Cell Tracking of cCFU-F

Live cell imaging was performed using a Leica live cell imaging microscope (DMI6000B) equipped with x-y-z controller and hardware autofocus. Phase contrast (PH) images were acquired every 15 minutes for 96 hours. GFP was detected every 2–3 hours (300ms exposure). Images were exported to Matlab to remove background noise, enhance contrast, and stitch contiguous sites. Custom-written software implemented in Matlab (Nordon’s Tracking Tool) was used to manually track cell nuclei through consecutive frames, to build trajectories and record fate outcomes (division, death, and right censored [lost or not complete]). Positions of cell nuclei in PH images were used to measure GFP intensity in the fluorescence channel, by recording pixel intensities. Two wells per condition were tracked.

### Animal ethics

All experimental methods involving mice were carried out in accordance with the relevant guidelines and regulations, and were approved by the St Vincent’s Hospital/Garvan Institute of Medical Research Animal Ethics Committee (AEC). AEC approval number 13/02.

### Statistical analysis

All statistical analyses were implemented in custom-written scripts in R^41^ and MATLAB^®^ (MATLAB 2015b, The MathWorks Inc., Natick, MA). The *timereg*^42^ and *mets* packages were used to implement competing risks regression and concordance analysis. A two-tailed Student’s *t* test (α = 0.05) was used to compare mean cell cycle times of BC cells. Details of all statistical tests applied are available as Supplementary material. All relevant code and cell lifetime data are available at the GitHub repository (https://github.com/Jamcor/crpaper).

## Additional Information

**How to cite this article**: Cornwell, J. A. *et al*. Quantifying intrinsic and extrinsic control of single-cell fates in cancer and stem/progenitor cell pedigrees with competing risks analysis. *Sci. Rep*. **6**, 27100; doi: 10.1038/srep27100 (2016).

## Supplementary Material

Supplementary Information

## Figures and Tables

**Table 1:** Comparison of statistical methods applied to single cell lifetime data.

Observations	Method	Selection bias	Stochastic model	Ref.
Time to fate	Mean time to fate: Group comparisons two-tailed unpaired Student *t*-test.	Excludes right censored lifetimes and CR	Not applicable	Rodgers *et al*.^43^
	Frequency distribution: Fitting of parametric distributions. Tests for goodness-of-fit		Parametric survival function	Duffy *et al*.^17^
	Kaplan Meier statistic: Cox regression analysis	CR are assumed to be right censoring times.	Empirical or semiparametric survival function with covariates	Huang *et al*.^14^
Counting fate outcomes	Frequency distributions and pie charts: Contingency tables and Pearson’s chi-squared test	Excludes time to fate and right censored lifetimes	Not applicable	Hallett *et al*.^20^
Time to fate and outcome	Pearson’s correlation coefficient: Fitting parametric multivariate distributions.	Excludes right censored lifetimes. Independent CR.	Temporal competition between uncorrelated pre-competition CR distribution	Duffy *et al*.^17^
	CR cumulative incidence function: CR regression	Includes right censored lifetimes and competing fates. Independent or dependent CR.	Non-parametric and semiparametric CIF for each fate with covariates	Not applied
Counting fate outcomes in kin	Yule’s Q, Binomial test: Concordance/discordance analysis	Excludes time to fate and right censored lifetimes	Not applicable	Scherf *et al*.^12^ Duffy *et al*.^17^
Time to fate and outcome in kin	Pearson’s correlation coefficient : Bivariate correlation analysis	Excludes right censored lifetimes and competing		Kinjyo *et al*.^30^
	Intraclass correlation coefficient (ICC), Pearson’s correlation coefficient (PCC).	Excludes right censored lifetimes and competing fates.	Monte Carlo simulation of kin lifetime assuming no concordance/discordance	Scherf *et al*.^12^
	Cross-odds ratio (COR): CR regression with clustering.	Includes right censored lifetimes and competing fates. Independent or dependent CR. Time invariant COR.	Gamma copula model to calculate conditional concordance probability of kin	Not applied

**Table 2:** Effect of kinship relationships on division and differentiation (GFP expression) concordance.

CRR model	Link function*	GFP**	Growth factor	log(COR) ±SE	p-value
*division CIF* ~ 1 + *const* (*GF*) *cluster* (*siblings*)	FG	−	MCSF/GCSF	20.9 ± 0.613	0
*division CIF* ~ 1 + *const* (*GF*) *cluster* (1^*st*^ *cousins*)	FG	−	MCSF/GCSF	3.68 ± 0.901	4 × 10^−5^
*division CIF* ~1 + *const* (*GF*) *cluster* (2^*nd*^ *cousins*)	FG	−	MCSF/GCSF	3.04 ± 0.629	1 × 10^−6^
*division CIF* ~ 1 + *GF* + *cluster* (*mother* – *daughter*)	FG	−	MCSF/GCSF	0.526 ± 0.158	9 × 10^−4^
*division CIF* ~ 1 + *cluster* (*sisters*)	FG	+	MCSF	3.71 ± 0.657	2 × 10^−8^
*division CIF* ~ 1 + *cluster* (1^*st*^ *cousins*)	FG	+	MCSF	2.13 ± 0.203	0
*division CIF* ~ 1 + *cluster* (2^*nd*^ *cousins*)	FG	+	MCSF	1.38 ± 0.116	0
*division CIF* ~ 1 + *cluster* (*mother* – *daughter*)	FG	+	MCSF	−0.19 ± 0.07	0.01
*GFP onset CIF* ~ 1 + *GF* + *cluster* (*siblings*)	Add	− → +	MCSF/GCSF	1.32 ± 0.345	2 × 10^−4^
*GFP onset CIF* ~1 + *cluster* (1^*st*^ *cousins*)	Add	− → +	MCSF/GCSF	3.36 ± 0.228	0
*GFP onset CIF* ~1 + *cluster* (2^*nd*^ *cousins*)	Add	− → +	MCSF/GCSF	2.91 ± 0.155	0
*GFP onset* ~ 1 + *GF* + *cluster* (*mother* – *daughter*)	Add	− → +	MCSF/GCSF	−0.935 ± 0.153	9 × 10^−10^

*FG = Fine – Gray model, Add = Additive model,

**− denotes GFP^−^, + denotes GFP^+^, (− → +) denotes a GFP negative to GFP positive transition.
