# Supplementary material for: Quantifying intrinsic and extrinsic control of single-cell fates in cancer and stem/progenitor cell pedigrees with competing risks analysis
Source: Sci Rep. 2016 Jun 1;6:27100. doi: 10.1038/srep27100 (PMC4890426; doi:10.1038/srep27100)
Supplement: Supplementary Information [file srep27100-s1.pdf]

Quantifying intrinsic and extrinsic control of single-cell fates in cancer and stem/progenitor cell pedigrees with competing risks analysis

JA Cornwell[1,2,3], RM Hallett[4], S Auf der Mauer[1], A Motazedian[4,5,6], T Schroeder[7], JS Draper[4,5,8], RP Harvey[2,3,9,10,*] and RE Nordon[1,3,*]

[1]*Graduate School of Biomedical Engineering, University of New South Wales, Sydney, NSW 2052, Australia*

[2]*Developmental and Stem Cell Biology Division, Victor Chang Cardiac Research Institute, Sydney, NSW 2010, Australia*

[3]*Australian Research Council Special Research Initiative in Stem Cell Science – Stem Cells Australia*

[4]*Department of Biochemistry and Biomedical Sciences, McMaster University, Hamilton, ON L8N 3Z5, Canada*

[5]*McMaster Stem Cell and Cancer Research Institute, Michael G DeGroote School of Medicine. Hamilton, Ontario, Canada*

[6]*Murdoch Children's Research Institute, The Royal Children's Hospital, Parkville, Victoria 3052, Australia*

[7]*Cell Systems Dynamics, Department of Biosystems Science and Engineering, ETH Zurich, Basel, Switzerland*

[8]*Department of Pathology and Molecular Medicine, McMaster University, Hamilton, ON L8N 3Z5, Canada*

[9]*St. Vincent's Clinical School, Faculty of Medicine, University of New South Wales, Sydney, NSW 2052, Australia*

[10]*School of Biotechnology and Biomolecular Sciences, University of New South Wales, Sydney, NSW 2052, Australia*

*Co-corresponding authors:

Robert Nordon
Graduate School of Biomedical Engineering,
University of New South Wales. Sydney, NSW 2052, Australia.
Email: r.nordon@unsw.edu.au

Richard Harvey
Victor Chang Cardiac Research Institute
Sydney, NSW 2052, Australia
Email: r.harvey@victorchang.edu.au

Table of contents

1. Application of competing risks statistics to cell lifetime data 1

   a) Estimation of empirical probability of a competing risk 2

   b) Testing group difference 2

   c) Competing risks regression (CRR) 2

   d) Cell clustering and concordance analysis 4

2. Development of semi-parametric models to study BC cell line chemotherapy resistance 5

   a) Development of semi-parametric CRR models 5

3. CR and concordance probability analysis of GMP data 8

4. Factorial experimental design to test effects of cytokines on cCFU-F growth dynamics 10

   a) Factorial analysis of colony formation and proliferation data 11

5. CR analysis of cCFU-F lifetime data 12

   a) Effects of cytokines on division and death 12

   b) Interaction effect of *Pdgfra*-GFP expression and cytokine stimulation on division and death 15

   c) Effect of *Pdgfra*-GFP expression and cytokine treatment on renewal of GFP^{+} cells 19

   d) Generation of GFP^{+} and GFP^{-} daughters from GFP^{+} and GFP^{-} mothers 20

   e) Concordance analysis of Pdgfra-GFP^{+} siblings 22

   f) Flow cytometry analysis of MSC surface markers 23

6. Permutation and randomization tests for cCFU-F sibling cell fate outcomes 24

7. References 25

1. Application of competing risks statistics to cell lifetime data

There is a substantial amount of literature describing the application of competing risks survival analysis to clinical trial data [1-5]. This has been driven by the need for tractable and rigorous methods to analyse patient-lifetime data, since the results of such analyses inform epidemiological-based health policy. While there are several commonly used survival statistics used by clinicians, including Kaplan-Meier analysis and Cox regression, it has been clearly demonstrated that such approaches will yield severely biased results in the presence of competing risks [4,6].

Competing risks (CR) are defined as any mutually exclusive fate outcomes that may occur during an individual's lifetime. An individual's lifetime is said to be 'right censored' if none of the competing outcomes are observed during the observation period. In a clinical trial, competing risks occur when there is more than one clinical endpoint for a patient (e.g., response to therapy, death from unrelated causes). Importantly, clinicians utilise CR analysis to quantify which treatment conditions may increase the probability of patient survival, or may lead to a higher probability of death. The scope of this paper does not include a detailed review of CR statistics, and therefore readers are encouraged to read the reviews highlighted above to understand the fundamental importance of these statistics in the clinical context.

In this manuscript, we adapt CR statistics to analyse cell lifetime data which are analogous to patient-lifetime data. An individual cell may have mutually exclusive fate outcomes (e.g. division and death); and a cell's lifetime may be right-censored (e.g. because it was lost during tracking or because its final fate was not observed before the end of the observation period).

In addition to using CR analysis to determine the effect of different treatment conditions on patient outcomes, Scheike et al. also developed CR concordance statistics to study the effect of inheritance on lifetime events in monozygotic and dizygotic twins [7]. For example, it was found that there was strong concordance in lifetime events such as menopause and breast cancer in monozygotic, but not dizygotic twins. Similarly, in this manuscript CR concordance statistics are applied to study the effect of inheritance on fate outcomes in cellular kin, i.e. the equivalent of monozygotic and dizygotic twins. Interestingly, single cell pedigrees provide an opportunity to study the effect of inheritance on fate outcomes over multiple generations, i.e. as genetic similarity progressively diverges. Such analyses provide an opportunity to determine the strength of latent determinants of cell fate by studying concordance in siblings, 1st cousins, and 2nd cousins, respectively.

The following sections describe the CR statistical methods that are applied in this manuscript. These include a) estimation of empirical probability of a competing risk; b) testing group difference; c) competing risks regression; and d) cell clustering and concordance analysis.

All of the described CR statistics are implemented using R-studio statistical software. For a detailed explanation of competing risks statistics and implementation in R see Scheike et al. [8], as well as the R packages required for implementation of CR

statistics including *timereg* [9], *survival,* and *mets*. All of the analysis code and data presented in this manuscript are available at the following GitHub repository https://github.com/Jamcor/crpaper. Any questions regarding code, analysis methods, and data format may be directed to the corresponding author.

a) Estimation of empirical probability of a competing risk

The *cumulative incidence* for competing risks was first described by Gray [10]. Cumulative incidence is an empirical estimate (non-parametric) of the probability (Pr) that a cell fate outcome (e.g. division or death) has occurred by a specified time or cell age(t). The cumulative incidence function (CIF) for event of type j in group k is defined as:

$$F_{jk}(t) = Pr[T_{ik} \leq t, \delta_{ik} = j]$$

where T_{ik} is the event time for individual i in group k, and δ_{ik} indicates the event type j. Observed experimental outcomes T_{ik} and δ_{ik} are random variates. The *cuminc* function in R was used to estimate empirical cumulative incidence functions.

b) Testing group difference

To test if there is a difference between experimental groups indexed by $k \in \{1, \ldots, K\}$ Gray developed a *K*-sample test for comparing the cumulative incidence of a competing risk. The null hypothesis for this test states that groups are independent and identically distributed $\left(H_0: F_{j1} = F_{j2} = \cdots = F_{jK}\right)$, though risks need not be independent as previously posited [11]. For example, we used this test to determine the effect of p53 mutation on the resistance of breast cancer cell lines to chemotherapy (Supplementary Text 2); competing event types were division $(j = 1)$ and death $(j = 2)$, and the index $k = 1 \ldots K$ denoted the chemotherapy treatment groups (Dox and Nut) and genotype (WT or MUT p53). The *ksample* function in R was used to test for group differences as described above.

c) Competing risks regression (CRR)

The development of non-parametric and semi-parametric CRR models followed the method described by Schieke and Zhang [12]. The goal was to select CRR models that are parsimonious (lowest number of estimated coefficients) and give rise to monotonically increasing CIFs. Therefore, semi-parametric models were preferable because fewer coefficients require estimation compared to non-parametric models. The development of semi-parametric CRR models was an iterative approach that required trial and error to select an optimal link function for the model. The *comp.risk* function was used to generate CRR models. Scheike and Zhang [8] described the development of flexible CRR models using the *comp.risk* function (contained within the *timereg* package). This manuscript applied the same approach for developing CRR models to analyse the effect of intrinsic and extrinsic factors on single-cell fate outcomes.

Briefly, semi-parametric models were fitted by 1) commencing with a non-parametric model and an arbitrary link function; 2) testing each covariate for time-invariant effects (using the Kolmogorov-Smirnov test included within the output of the

comp.risk function); 3) if non-parametric covariates were time invariant (p>>0.05) they were substituted with parametric covariates (see below); and 4) steps 2-3 were repeated using different link functions (proportional, additive, Fine and Gray) to find the link function that maximised the number of parametric terms; while checking that the CIF is monotonically increasing (sometimes the additive link function gave rise to decreasing CIFs). For readers with a background in statistics, a mathematical description of this procedure is provided below.

Fine and Gray developed a proportional hazards model to quantify the effect of one or more covariates on competing risks [13]. For cause $j = 1$, the cumulative incidence function is conditional on covariates, $\boldsymbol{Z}$, a vector denoting one or more cell extrinsic or intrinsic covariates that may influence cell fate: $F_1(t;\boldsymbol{Z}) = Pr[T \leq t, \delta = 1|\boldsymbol{Z}]$. The subdistribution hazard for cause $j = 1$ as defined by Gray is $\lambda_1(t;\boldsymbol{Z}) = \{dF_1(t;\boldsymbol{Z})/dt\}/\{1 - F_1(t;\boldsymbol{Z})\}$ the probability density of the subdistribution divided by the complement of the subdistribution. A non-parametric baseline hazard function $\lambda_{10}(t)$ and parameter vector β_0 are estimated using a Cox-like regression model $\lambda_1(t;\boldsymbol{Z}) = \lambda_{10}(t)\exp\{\boldsymbol{Z}^T\beta_0\}$ where the term $\exp\{\boldsymbol{Z}^T\beta_0\}$ is interpreted as fold increase in risk (from baseline) given covariate $\boldsymbol{Z}$.

Scheike et al. have since developed more flexible semi-parametric competing risk regression models [12], which are applied when the Fine and Gray model does not fit the data well. They consider a class of general regression models

$$h\{F_1(t;\boldsymbol{Z})\} = g\{\boldsymbol{\eta}(t), \boldsymbol{\gamma}, \boldsymbol{Z}\}$$

h and g are known link function and regression functions, and $\boldsymbol{\eta}(t)$ and γ are unknown regression functions and parameters, respectively. The covariates $\boldsymbol{Z} \equiv [\boldsymbol{x}, \boldsymbol{z}]$ can be split between non-parametric $\boldsymbol{\eta}(t)$ and parametric $\boldsymbol{\gamma}$ terms of the regression model. They focus on two classes of models, the semiparametric multiplicative (proportional) model $h\{F_1(t;\boldsymbol{Z})\} = \log\{-\log(1 - F_1(t;\boldsymbol{Z}))\}$, $g\{\boldsymbol{\eta}(t), \gamma, \boldsymbol{Z}\} = \boldsymbol{\eta}(t)^T\boldsymbol{x} + \boldsymbol{\gamma}^{\boldsymbol{T}}\boldsymbol{z}$, and the semiparametric additive model $h\{F_1(t;\boldsymbol{Z})\} = -\log(1 - F_1(t;\boldsymbol{Z}))$ $g\{\boldsymbol{\eta}(t), \gamma, \boldsymbol{Z}\} = \boldsymbol{\eta}(t)^T\boldsymbol{x} + (\boldsymbol{\gamma}^{\boldsymbol{T}}\boldsymbol{z})t$. When $\boldsymbol{z} = \boldsymbol{0}$ the model is nonparametric. The multiplicative model simplifies to the Fine and Gray model when $\boldsymbol{x} = 1$.

Scheike developed a goodness-of-fit procedure based on a statistical test for the hypothesis that non-parametric coefficients for the regression model are constant over time. Starting with a fully non-parametric model where $\boldsymbol{z} = \boldsymbol{0}$, the number of estimated coefficients and model complexity is reduced iteratively by substituting the jth non-parametric term $\eta_j(t)x_j$ with a parametric term $\gamma_j z_j$ (multiplicative) or $\gamma_j z_j\, t$ (additive) if $\eta_j(t)$ is considered to be constant over time. A constant (time invariant) non-parametric effect is identified by testing the null hypothesis by the Kolmogorov-Smirnov test ($\eta_j(t) = \eta_j$; multiplicative model or $\eta_j(t) = \eta_j t$; additive model). The output of the R function comp.risk (*timereg* package, version 1.7.0, [9]) displayed the result of this test as the '*Test for time invariant effects',* where the probability value is given by *p-value H_0:constant effect*. If the test is non-significant (p>0.05) one assume that the null hypothesis is true, and the model can be simplified by assuming a parametric term for covariate *j*.

The significance of non-parametric covariates was tested using the Supremum-test of significance and was output by the *comp.risk* function under the heading '*Test for non-significant effects'* (i.e. $p<0.05$ taken as a significant effect). Parametric covariates, their standard error and level of significance are output if the model has parametric terms.

The goodness-of-fit of the semi-parametric CRR model was also inspected by comparison with the non-parametric model, and the empirical Gray subdistribution for those covariates where possible (*cuminc* function, *cmprsk* R package) [10].

Competing risks regression (CRR) models are constructed using R shorthand notation that include specific terms. Firstly, a baseline CIF is indicated by the presence of a number one (1) to the right of a tilde (~). To the right of the baseline CIF any number of terms may be included in the model separated by a plus sign (+). Parametric terms are identified by wrapping them with *const()*. E.g. if A was a parametric term it would be included as const(A) in the model. Models can also include R shorthand notation to represent the nth order interactions ($n = 1,2, or\ 3$):

$$Event\ CIF \sim 1 + \ (a + b + c)^n$$

For example if $n = 2$, then $(a + b + c)^2 \equiv a + b + c + a{:}b + a{:}c + b{:}c$.

After construction of the model terms the *comp.risk* function requires selection of a link function (as described above) and specification of the fate outcomes being evaluated (specified by a coded cause number). Link functions may either additive (*additive*), proportional (*prop*), or a Fine and Gray (*fg*). In all the models presented here the fate outcomes (causes) are coded as 0 for right-censored, 1 for division, and 2 for death.

d) Cell clustering and concordance analysis

Scheike at al. proposed a cross-odds ratio (COR) function to measure the association of cause-specific event times within a cluster [7]. In the cellular context, a cluster is defined as any pair of cells that share a common ancestor and are separated by the same familial distance, i.e. are in the same generation (Figure S1). For example, clusters included sister pairs, mother and daughter pairs, and cousins (1st, 2nd, 3rd, etc). Importantly, when evaluating concordance for kinship pairs multiple counting of the same cell was prevented by randomly sampling kinship clusters without counting the same cell twice.

The COR was used to determine if a cell's fate is correlated with the fate of the other cell within the cluster (i.e. does the fate outcome for one sibling in a sibling cluster depend on the fate of its sibling). Therefore, the COR can provide evidence that a cell's fate is influenced by heritable determinants of cell fate. If cell fates are dependent within a cluster, a significant value of the cross odds ratio indicates whether these fates are concordant (symmetric) or discordant (asymmetric).

The odds of event *A* is defined as the probability of event *A* divided by the probability of its complement:

$$\mathrm{ODDS}[A] = \frac{\Pr[A]}{1 - \Pr[A]}$$

The conditional odds of event *A* given *B* is defined

$$\mathrm{ODDS}[A|B] = \frac{\Pr[A|B]}{1 - \Pr[A|B]}$$

And the cross odds ratio is

$$\mathrm{XODDS}[A, B] = \frac{\mathrm{ODDS}[A|B]}{\mathrm{ODDS}[A]}$$

A is the occurrence in cell i belonging to cluster *k* of the competing event e_i occurring before time *t* $[A \stackrel{\text{def}}{=} \{T_{ki} \leq t,\} \cap \{\varepsilon_{ki} = e_i\}]$ where T_{ki} and ε_{ki} are random variables denoting competing event time and cause, respectively. Likewise, *B* is the occurrence in cell j belong to cluster *k* of competing event e_j occurring before time *t* $\left[A \stackrel{\text{def}}{=} \left\{T_{kj} \leq t,\right\} \cap \left\{\varepsilon_{kj} = e_j\right\}\right]$.

If events within the cluster are independent then $\mathrm{ODDS}[A] = \mathrm{ODDS}[A|B]$ and $\mathrm{XODDS}[A, B] = 1$. If events are concordant then the occurrence of event *B* increases the odds of event *A*, so $\mathrm{XODDS}[A, B] > 1$. If cells are sibling pairs then synchronous events have a COR that is significantly greater than 1. If event are discordant, then the occurrence of event *B* decreases the odds of event *A,* so $0 < \mathrm{XODDS}[A, B] < 1$. A cross odds ratio that is significantly less than 1 indicates asymmetric fates that could not occur by chance alone.

In this manuscript the degree of concordance of cells within a cluster was visualised by plotting the cumulative distributions $\Pr[A]$ and $\Pr[A|B]$ (probandwise concordance). If $\Pr[A|B]$ are within the confidence interval of $\Pr[A]$ then one has less confidence that cause-specific events within a cluster are dependent. Thus, one needs to compare the conditional distribution $\Pr[A|B]$ with the unconditional distribution $\Pr[A]$ to test whether symmetric or asymmetric events occur by chance alone.

2. Development of semi-parametric models to study BC cell line chemotherapy resistance

The *comp.risk* function from the *timereg* package [12] in R was used to fit non-parametric and semi-parametric regression models to BC cell lifetime data. Development of semi-parametric CRR models was desirable because they estimated coefficients (one for each covariate) that described how the probability of a cell fate outcome was modified by a covariate [12].

a) Development of semi-parametric CRR models

Briefly, semi-parametric models were fitted by 1) selecting a link function; 2) testing each covariate for time-invariant effects (using the Kolmogorov-Smirnov test); and 3) estimating parameters for time-invariant effects and empirical coefficients. The link function scaled the empirical baseline CIF to link it to other CIFs using estimated

parametric and empirical coefficients. Constant coefficients were estimated for covariates that passed the time invariance test ($p>0.05$), otherwise an empirical function was used to model time-variant effects. For example, a semi-parametric CIF for division was estimated using an additive link function, effects of the covariates WT, WT:Nut, and WT:Dox were represented by constant coefficients (Table S1- S3). Effects of Nut and Dox were estimated using empirical functions because they failed the test for time invariance ($p<0.05$). Goodness-of-fit was assessed by comparing semi-parametric and non-parametric CIFs (Fig. 2c-d, solid versus dashed lines).

The non-parametric model shown in equation (S2.1) was used to estimate the effect of p53 mutations on BC cell resistance to chemotherapy:

Equation S2.1

$$division\ (or\ death) \sim 1 + WT + Dox + Nut + WT{:}Dox + WT{:}Nut$$

Each covariate was tested for time invariance using the Kolmogorov-Smirnov (KS) test (Supplementary Table S1-2). If the null hypothesis (H0) is rejected ($p<0.05$) it means that the test for time invariance has failed, so a non-parametric model to describe the time variant effects of that covariate is required. The covariates where the KS test does not fail can be modelled using a constant parameter.

Supplementary Table S1 | Results from the Kolmogorov-Smirnov test for time invariance applied to the model in equation (S2.1). The results from this test were used to define constant ($p>0.05$) and time-variant ($p<0.05$) effects on BC cell division.

**Covariate**	**Additive link function**	
	**Kolmogorov-Smirnov test**	**p-value (H0: constant effect)**
**Baseline CIF**	0.616	0
**WTp53**	0.561	0.142
**Dox**	0.605	0
**Nut**	0.793	0
**WTp53:Dox**	0.393	0.582
**WTp53:Nut**	0.52	0.196

All time invariant non-parametric covariates were substituted with parametric covariates to develop a semi-parametric model for division as shown in, as shown in equation (S2.2).

Equation S2.2

$$\text{division CIF} \sim 1 + \text{const(WT)} + \text{Dox} + \text{Nut} + \text{const(WT: Dox)} + \text{const(WT: Nut)}$$

where *const*( ) is used to denote parametric covariates. The estimated coefficients for division are shown in Table S3.

This model was then used to obtain CIFs for any factor, or combination of factors, by adding their effects to the baseline CIF. For example, non-parametric CIFs for division and death of WT cells exposed to Nut were predicted by substituting WT = 1, Dox = 0, Nut = 1, WT:Dox = 0 (representing the interaction between WT and Dox), and WT:Nut = 1 into the model.

Supplementary Table S2 | Results from the Kolmogorov-Smirnov test for time invariance applied to the model in equation (S2.1). The results from this test were used to define constant (p>0.05) and non-constant (p<0.05) effects on BC cell death.

**Covariate**	**Additive link function**	
	**Kolmogorov-Smirnov test**	**p-value (H0: constant effect)**
**Baseline CIF**	0.0136	0.244
**WTp53**	0.0125	0.646
**Dox**	0.223	0
**Nut**	0.0474	0.198
**WTp53:Dox**	0.0558	0.182
**WTp53:Nut**	0.204	0.004

All time invariant non-parametric covariates were substituted with parametric covariates to develop a semi-parametric model for death as shown in, as shown in equation (S2.3).

Equation S2.3

$$\text{death CIF} \sim 1 + \text{const(WT)} + \text{Dox} + \text{const(Nut)} + \text{const(WT: Dox)} + \text{WT: Nut}$$

where *WT*, *Nut*, and *WT:Dox* had constant (time invariant) coefficients. The estimated coefficients for death are shown in Table S3.

Supplementary Table S3 | Results from semi-parametric models (shown in equation (S2.2) and equation (S2.3)) describing the effect of Nut and Dox on division and death outcomes in mutant and wild-type BC cell lines*

Effect		Probability relative to mutant p53 breast cancer (±SE)	
**Intrinsic (p53)**	**Extrinsic**	**Division[1]**	**Death[2]**
WT	nil	0.63±0.20 ($p<0.005$)	-0.0034±0.0088 (NS)
MUT	Nut	negative ($p<0.01$)	-0.017±0.013 (NS)
MUT	Dox	negative ($p<0.001$)	positive ($p<0.001$)
WT	Nut	-1.15±0.22 ($p<0.001$)	-0.005±0.016 (NS)
WT	Dox	-0.58±0.20 ($p<0.005$)	negative ($p<0.005$)

Semi-parametric models (see Supplementary Text 1c)

1. Division CIF: ~1+const(WT) + Dox + Nut + const(WT*Nut) + const(WT*Dox). Additive link function.
2. Death CIF: ~1+const(WT)+Dox+const(Nut)+const(WT*Nut)+wt:Dox. Additive link function.

3. CR and concordance probability analysis of GMP data

To study concordance in the fate of cellular kin in GMP and their differentiated progeny we applied CR concordance probability statistics (see Text 1c).

First we constructed a non-parametric CRR model to test for the effects of growth factor (GF) treatment on the probability of division and death for GMPs treated with either MCSF or GCSF. The non-parametric CRR model is shown in equation (S3.1).

Equation S3.1

$$division\ (or\ death) CIF \sim 1 + GFP + GF{:}GFP + GF$$

In this model we found that the GF term had no effect on division or death of GMPs until they differentiated into either macrophage or granulocyte progeny. Therefore, the GF term was excluded from the model.

We then investigated concordance in fate for sibling, mother-daughter, 1st cousin, and 2nd cousin clusters by constructing CRR models that included a term for each kinship cluster. See Table 2 in the main text for a description of the CRR models used. The cross-odds ratio (COR) was calculated using the *cor.cif* (contained within the *mets* R package) function using the symmetry condition (sym=1). The pseudo-code below shows the implementation of the *cor.cif* function in R.

```
cor.cif(cif = cif.model.division, data = tempdata, cause1 = 1,  cause2 = 1, sym = 1)
```

where *cif* was the output from the CRR models (Table 2, main text), *data* contained cell lifetimes for division, death, and right-censoring. cause1 and cause2 were the fate outcomes of interest for each cell in the kinship cluster, respectively. For example,

cause1=1 and cause2=1 allowed for the *cor.cif* function to compute the COR for sibling 1 division and sibling 2 division. The same method was used to estimate COR for mother-daughter pairs, 1st cousins, and 2nd cousins.

Yule's Q was used to quantify concordance in cell fate for undifferentiated (GFP^-) GMPs. The function *Yule* from the R package *psych* was used. To determine correlation in time to division for GFP^- cells the ICC and PCC were calculated. The function *ICCest* from the package *ICC* and the function *cor* from the package *stats* were used. The results for each of these tests are shown in Table S4. While these tests are routinely used to quantify association in cell fate outcomes, as shown in Table S4 they exclude large amounts of cell lifetime data.

Table S4 | Contemporary statistical tests used to quantify concordance in time to division for undifferentiated (GFP^-) GMPs. This table also includes a direct comparison of the percentage of cell lifetime data used by each statistical test.

Statistical test	Yule's Q	% cell lifetimes used	ICC (±95%CI)	% cell lifetimes used	Pearson's correlation	% cell lifetimes used	COR (±SE)	% cell lifetimes used
**Mother-daughter**	1	69.7	0.217 (0.023)	56.7	0.61	56.7	0.526 (0.158)	100
**Siblings**	0.688	66.6	0.596 (0.021)	52.3	0.957	52.3	20.9 (0.613)	100
**1st cousins**	0.721	66.1	0.526 (0.0196)	51.5	0.845	51.5	3.68 (0.901)	100
**2nd cousins**	0.744	62.9	0.514 (0.0169)	48.7	0.814	48.7	3.04 (0.629)	100
**Average (%)**	-	66.3	-	52.3	-	52.3	-	100

4. Factorial experimental design to test effects of cytokines on cCFU-F growth dynamics

To quantify the effect of individual cytokines and cytokine combinations on cCFU-F division, death, and self-renewal we employed factorial design experiments. Effects of cytokines on both cell number and CFU frequency were tested in population-based experiments. The effects of cytokines on the probability of division and death were assessed by single-cell tracking experiments. For all experiments two-level, full factorial design experiments were chosen since such designs have the power to quantify the effect of individual factors as well as combinations of factors. In the first design the cytokines tested and their levels were: FGF (0 and 4ng/ml), TGF (0 and 2ng/ml), and PDGF (0 and 50ng/ml). The design of this experiment is shown in Table S5. The cytokine concentrations were selected based on the result of dose response experiments measuring cCFU-F doubling time (data not shown), where the minimum concentration that gave maximum responses (shortest doubling time) were selected. Below is a description of how the results of factorial design experiments were analysed to quantify the effect of cytokines on cCFU-F growth dynamics.

Supplementary Table S5 | The design of the full factorial experiment used to test for effects of FGF, TGF, and PDGF on cCFU-F growth in serum free medium. (+) and (-) denote presence and absence of each factor, respectively.

Condition	PDGF	TGF	FGF
**No factors**	-	-	-
**PDGF**	+	-	-
**TGF**	-	+	-
**FGF**	-	-	+
**PDGF/TGF**	+	+	-
**PDGF/FGF**	+	-	+
**TGF/FGF**	-	+	+
**PDGF/TGF/FGF**	+	+	+

The effect of cytokines and their interactions on cell population data (CFU frequency and population doublings) was calculated from their contrasts and ANOVA as described in Montgomery's text [14]. CR regression analysis in section was used to estimate the effect of growth factors and their higher order interaction on the CIF for division or death. The regression model uses R shorthand notation to represent the nth order factor interactions ($n = 1,2, or\ 3$):

$$Event\ CIF \sim (a + b + c)^n$$

For example if $n = 2$, then $(a + b + c)^2 \equiv a + b + c + a{:}b + a{:}c + b{:}c$.

a) Factorial analysis of colony formation and proliferation data

Main effects are defined as a change in response (e.g. cell number, colony number, rate of division or death) as a consequence of a change in the level of a factor (e.g. an increase in FGF from 0ng/ml to 4ng/ml). As outlined above a two-level, full factorial design experiment measures the response and effect of all factor combinations. The main effect of factor A is calculated by subtracting the responses where factor A is absent from the responses where factor A is present. Interaction effects (e.g. interaction of factor A and B) are defined as the average difference between the effect of A at the high level of B and the effect of A at the low level of B. Therefore main and interaction effects were calculated using contrasts which can be defined for *k* factors using the formula,

$$Contrast_{AB\ldots K} = (a \pm 1)(b \pm 1)\ldots.(k \pm 1)$$

where lowercase letters are the sum of the response of replicate experiments. The sign in each parentheses is negative if a factor was present in the effect and positive if the factor was absent. For example, contrasts for factors A and B are

$$Contrast_A = ab + a - b - (1)$$

$$Contrast_B = ab + b - a - (1)$$

$$Contrast_{AB} = ab - a - b + (1)$$

where (1) represents responses where no factors are present. Using contrasts, effects can be calculated

$$Effect_{AB\ldots K} = \frac{2}{n2^k} Contrast_{AB\ldots K}$$

The total sum of squares, SS_{total}, can be partitioned into the sum of squares of main and interaction effects, $SS_{AB\ldots K}$, and the error sum of squares, SS_{error},

$$SS_{total} = \sum_{AB\ldots K} SS_{AB\ldots K} + SS_{error}$$

where,

$$SS_{total} = \sum_{j=1}^{n} \sum_{i=1}^{2^k} \left(y_{ij} - \bar{y}\right)^2, \qquad SS_{AB\ldots K} = (Contrast_{AB\ldots K})^2 / n2^k$$

and y_{ij} is the response of the *i*th cytokine combination and the *j*th replicate and $\bar{y}$ is the average response. Error sum of squares may be calculated from the difference between total sum of squares and sum of squares of effects. The F distribution and statistic were used to test for significance in treatment effects.

$$F = \frac{SS_{AB\ldots K}}{s^2}$$

Where each effect has a single degree of freedom and the mean squared error $s^2 = SS_{error} / 2^k(n-1)$ has $2^k(n-1)$ degrees of freedom. The null hypothesis (of no effect) was rejected if the probability of the effect was less than 0.05.

5. CR analysis of cCFU-F lifetime data

a) Effects of cytokines on division and death

Cells exposed to the conditions outlined in a factorial design experiment (Supplementary Table S5) were tracked for a period of 96 hours. For generation 0, the CR regression model for division is shown in equation (S5.1).

Equation S5.1

$$division\ CIF\ (generation\ \ 0) \sim 1 + \ (TGF + PDGF + FGF)^3$$

The R output for this non-parametric CR regression model (using the additive link function) is summarised in Supplementary Table S6 and Supplementary Table S7.

Supplementary Table S6 | Results from the Supremum-test of significance applied to the model shown in equation (S5.1). The results from this test were used to identify non -significant effects (p>0.05) of cytokines on cCFU-F division in generation 0 cells.

	Supremum-test of significance	p-value H_0: B(t)=0
**(Intercept)**	4.38	0
**TGF**	2.46	0.092
**PDGF**	3.3	0.006
**FGF**	3.48	0.002
**TGF:PDGF**	2.25	0.202
**TGF:FGF**	1.97	0.3
**PDGF:FGF**	2.58	0.082
**TGF:PDGF:FGF**	3.26	0.016

Supplementary Table S7 | Results from the Kolmogorov-Smirnov test for time invariance applied to the model in equation (S5.1). The results from this test were used to define constant (p>0.05) and non-constant (p<0.05) effects of cytokines on cCFU-F division.

	Kolmogorov-Smirnov test	p-value H_0:constant effect
**(Intercept)**	0.0489	0.61
**TGF**	0.228	0.592
**PDGF**	0.492	0.312
**FGF**	0.355	0.162
**TGF:PDGF**	0.654	0.332
**TGF:FGF**	0.298	0.902
**PDGF:FGF**	0.283	0.948
**TGF:PDGF:FGF**	1.36	0.082

Note that there were no significant time varying effects (KS>0.05) so a semi-parametric model was fitted to this data. const() indicates parametric terms. The R output for this semi-parametric model is shown in Supplementary Table S8.

Equation S5.2

$$division\ CIF\ (generation\ \ 0) \sim 1 + \left(const(TGF) + const(PDGF) + const(FGF)\right)^3$$

Supplementary Table S8 | Results from the semi-parametric model (shown in equation (S5.2)) describing the effect of cytokines on cCFU-F division for generation 0 cells.

Parametric covariate	Coefficient	SE	Robust SE	z	P-value
**const(TGF)**	0.249	0.112	0.112	2.22	0.0263
**const(PDGF)**	0.397	0.122	0.122	3.25	0.00115
**const(FGF)**	0.486	0.152	0.152	3.2	0.00137
**const(TGF:PDGF)**	-0.296	0.214	0.214	-1.39	0.166
**const(TGF:FGF)**	-0.395	0.253	0.253	-1.56	0.119
**const(PDGF:FGF)**	-0.52	0.213	0.213	-2.44	0.0147
**const(TGF:FGF:PDGF)**	2.55	0.759	0.759	3.36	0.00078

The effect of single growth factors on cells (generation 0) is shown in Supplementary Figure S5a as a quantile plot. These effects are consistent with empirical CIFs (produced using *cuminc*) for separate experimental groups with various growth factor combinations as shown in Supplementary Figure S5b.

A similar approach was used to construct a semi-parametric CRR model for cells whose birth was observed (generation > 0):

Equation S5.3

$$\text{division CIF (generation} > 0)\sim 1 + \text{const(TGF)} + \text{const(PDGF)} + \text{const(FGF)}$$

In this model the Fine and Gray link function was used. All model parameters were found to be time invariant (data not shown), however, higher order interactions were not included in this model because they were not significant (data not shown). The model showed that FGF and PDGF had significant effects on division of generation > 0 cells, while TGF did not (Supplementary Table S9). The CIFs for this model are shown in Supplementary Figure S5c.

Supplementary Table S9 | Results from the semi-parametric model (shown in equation (5.3)) describing the effect of cytokines on cCFU-F division for generation > 0 cells.

Parametric covariate	Coefficient	SE	Robust SE	z	P-value
**const(TGF)**	-0.007	0.181	0.181	-0.04	0.97
**const(PDGF)**	0.399	0.197	0.197	2.03	0.0427
**const(FGF)**	0.457	0.195	0.195	2.34	0.0192

A non-parametric model was developed to quantify the effect of cytokines on cell death in generation > 0 cells, as shown in equation (S5.4).

Equation S5.4

$$\text{death CIF (generation} > 0)\ \sim 1 + (\text{TGF} + \text{PDGF} + \text{FGF})^3$$

The results from this model showed that TGF-β1 was the only growth factor that increased the rate of cell death (Supplementary Figure S5d and Table S10).

Supplementary Table S10 Results from the Supremum-test of significance applied to the model shown in equation (S5.4). The results from this test were used to identify non -significant effects (p>0.05) of cytokines on cCFU-F death in generation > 0 cells.

	Supremum-test of significance	p-value H_0: B(t)=0
**(Intercept)**	4.44	0
**TGF**	5.02	0
**PDGF**	1	0.578
**FGF**	1.62	0.49
**TGF:PDGF**	1.3	0.506
**TGF:FGF**	1.42	0.5
**PDGF:FGF**	1.41	0.382
**TGF:PDGF:FGF**	1.51	0.454

Supplementary Table S11 | Results from the Kolmogorov-Smirnov test for time invariance applied to the model in equation (S5.4). The results from this test show constant ($p>0.05$) and non-constant ($p<0.05$) effects of cytokines on cCFU-F death in generation>0 cells.

	**Kolmogorov-Smirnov test**	**p-value H_0:constant effect**
**(Intercept)**	0.0636	0.152
**TGF**	0.0475	0.71
**PDGF**	0.081	0.11
**FGF**	0.079	0.138
**TGF:PDGF**	0.11	0.404
**TGF:FGF**	0.142	0.564
**PDGF:FGF**	0.116	0.038
**TGF:PDGF:FGF**	0.222	0.324

b) Interaction effect of *Pdgfra*-GFP expression and cytokine stimulation on division and death

This section describes the development of CRR models used to study the interaction of Pdgfra-GFP expression with cytokine treatment. Firstly, the fidelity of GFP as a reporter for PDGFRα expression and mitogenic activity was tested using a CRR model that included a term for GFP expression, as shown in equation (S5.5). GFP expression was measured at the end of a cell's lifetime, i.e. before division, death, or right-censoring (this is referred to as GFPAtDeath in the model below). GFP expression was a continuous variable that represent raw measurements of pixel intensity (arbitrary fluorescent units) after image processing (see Materials and Methods). The model also included terms for treatment with TGF-β1, PDGF or FGF.

Equation S5.5

$$\begin{aligned}\text{division CIF} \sim 1 + \ &\text{const(TGF)} + \text{const(PDGF)} + \text{const(FGF)} + \text{const(GFPAtDeath)} \\ &+ \text{const(GFPAtDeath} * \text{TGF)} + \text{const(GFPAtDeath} * \text{PDGF)} \\ &+ \text{const(GFPAtDeath} * \text{FGF)}\end{aligned}$$

There was a very strong interaction between GFPAtDeath (end of a cell's lifetime) and PDGF treatment, confirming that GFP expression was correlated with PDGF signalling activity (Table S12).

Supplementary Table S12 | Results from the semi-parametric model (shown in equation (S5.5)) describing the effect of cytokines and GFP expression (at the end of a cell's lifetime) on cCFU-F division for generation > 0 cells.

Parametric covariate	Coefficient	SE	Robust SE	z	P-value
**const(TGF)**	-0.0091	0.0443	0.0443	-0.206	8.37E-01
**const(PDGF)**	-0.0891	0.0416	0.0416	-2.14	3.23E-02
**const(FGF)**	0.0727	0.0484	0.0484	1.5	1.33E-01
**const(GFPAtDeath)**	0.00034	0.0004	0.00044	0.782	4.34E-01
**const(GFPAtDeath:PDGF)**	0.0012	0.0003	0.000257	4.68	4.28E-01
**const(GFPAtDeath:TGF)**	0.00027	0.0003	0.000341	0.792	2.91E-06
**const(GFPAtDeath:FGF)**	-0.0003	0.0004	0.000436	-0.69	4.90E-01

A similar model was developed to investigate the effect of GFP expression as measured at cell birth (GFPAtBirth), shown in equation (S5.6).

Equation S5.6

$$\text{division CIF} \sim 1 + \text{const(TGF)} + \text{const(PDGF)} + \text{const(FGF)} + \text{const(GFPAtBirth)} + \text{const(GFPAtBirth} * \text{TGF)} + \text{const(GFPAtBirth} * \text{PDGF)} + \text{const(GFPAtBirth} * \text{FGF)}$$

There was no interaction between GFP fluorescence at cell birth (measured immediately after mitosis) and PDGF suggesting that changes GFP fluorescence just before mitosis more closely correlated with PDGFRα mitogenic activity (Supplementary Table S13).

Supplementary Table S13 | Results from the semi-parametric model (shown in equation (S5.6)) describing the effect of cytokines and GFP expression (at cell birth) on cCFU-F division for generation > 0 cells.

Parametric covariate	Coefficient	SE	Robust SE	z	P-value
**const(TGF)**	-3.48E-03	0.0501	0.0501	-0.0695	0.945
**const(PDGF)**	1.09E-01	0.0454	0.0454	2.4	0.0165
**const(FGF)**	1.72E-01	0.0424	0.0424	4.05	0.000051
**const(GFPAtBirth)**	-1.99E-04	0.000214	0.000214	-0.93	0.352
**const(GFPAtBirth:PDGF)**	-1.23E-04	0.000294	0.000294	-0.42	0.675
**const(GFPAtBirth:TGF)**	-5.05E-04	0.000386	0.000386	-1.31	0.191
**const(GFPAtBirth:FGF)**	4.49E-05	0.000213	0.000213	0.211	0.833

We also developed a CRR model used to quantify effects of GFP expression and cytokine treatment on cell death, shown in equation (S5.7).

Equation S5.7

$$\text{death CIF} \sim 1 + \text{const(TGF)} + \text{const(PDGF)} + \text{const(FGF)} + \text{const(GFPAtDeath)} + \text{const(GFPAtDeath} * \text{TGF)} + \text{const(GFPAtDeath} * \text{PDGF)} + \text{const(GFPAtDeath} * \text{FGF)}$$

The results produced by this model showed that nuclear GFP intensity at the end of a cell's lifetime was not related to the rate of cell death (Supplementary Table S14).

Supplementary Table S14 | Results from the semi-parametric model (shown in equation (S5.7)) describing the effect of cytokines and GFP expression (at the end of a cell's lifetime) on cCFU-F death for generation > 0 cells.

Parametric covariate	Coefficient	SE	Robust SE	z	P-val
**const(TGF)**	6.18E-02	2.35E-02	2.35E-02	2.63	0.00848
**const(PDGF)**	-2.01E-02	1.83E-02	1.83E-02	-1.1	0.273
**const(FGF)**	2.82E-02	2.43E-02	2.43E-02	1.16	0.246
**const(GFPAtDeath)**	1.41E-04	1.77E-04	1.77E-04	0.796	0.426
**const(GFPAtDeath:PDGF)**	-1.92E-05	1.96E-05	1.96E-05	-0.98	0.327
**const(GFPAtDeath:TGF)**	-1.48E-05	1.00E-04	1.00E-04	-0.148	0.882
**const(GFPAtDeath:FGF)**	-1.59E-04	1.78E-04	1.78E-04	-0.889	0.374

In order to classify cells as GFP^+ and GFP^- we established a threshold for GFP intensity below which there was no effect of PDGF. Using this method, a threshold of 100 fluorescent units defined PDGFRα^+ (GFP^+) and PDGFRα^- (GFP^-) cell subsets. We fitted a non-parametric CR regression model, shown in equation (S5.8), using this classification for PDGFRα expression. In this model, GFP is a categorical variable (GFP=0 represented GFP^- cells and GFP=1 represented GFP^+ cells).

Equation S5.8

$$\text{division CIF} \sim 1 + (\text{GFP} + \text{PDGF})^2$$

The terms in the model were tested for non-significant effects (Supplementary Table S15) and time- invariance with respect to the covariates GFP, PDGF and PDGF:GFP (Supplementary Table S16) which enabled the construction of a semi-parametric model, shown in equation (S5.9).

Equation S5.9

$$\text{division CIF} \sim 1 + (\text{const(GFP)} + \text{const(PDGF)})^2$$

The semi-parametric model showed that GFP and PDGF alone did not have significant effects on the probability of division, however, the interaction GFP:PDGF was significant (Supplementary Table S17). The CIFs derived from this model are shown in Figure 4d.

Supplementary Table S15 | Results from the Supremum-test of significance applied to the model shown in equation (S5.8). The results from this test were used to identify non -significant effects (p>0.05) of cytokines and GFP expression on cCFU-F division in generation > 0 cells.

	Supremum-test of significance	p-value H_0: B(t)=0
**(Intercept)**	3.67	0.002
**GFP**	2.27	0.228
**PDGF**	1.73	0.52
**GFP:PDGF**	4.77	0

Supplementary Table S16 | Results from the Kolmogorov-Smirnov test for time invariance applied to the model in equation (S5.8). The results from this test show constant (p>0.05) and non-constant (p<0.05) effects of cytokines and GFP expression on cCFU-F division in generation>0 cells.

	Kolmogorov-Smirnov test	p-value H_0: B(t)=0
**(Intercept)**	0.623	0.000
**GFP**	0.684	0.170
**PDGF**	0.167	0.634
**GFP:PDGF**	2.740	0.456

Supplementary Table S17 | Results from the semi-parametric model (shown in equation (S5.9)) describing the effect of cytokines and GFP expression on cCFU-F division for generation > 0 cells.

Parametric covariate	Coefficient	SE	Robust SE	z	P-value
**const(GFP)**	0.47	0.337	0.337	1.4	0.163
**const(PDGF)**	-0.232	0.325	0.325	-0.712	0.476
**const(GFP):const(PDGF)**	1.1	0.417	0.417	2.63	0.0085

c) Effect of *Pdgfra*-GFP expression and cytokine treatment on renewal of GFP$^+$ cells

To study self-renewal of GFP$^+$ cells we divided possible fate outcomes into four CR categories.

1) GFP$^+$ division
2) GFP$^-$ division
3) GFP$^+$ death
4) GFP$^-$ death

These categories were used to identify the fate outcome of interest in the CRR model shown in equation (S5.10).

Equation S5.10

$$\text{division CIF} \sim 1 + \text{const(PDGF)} + \text{const(FGF)}$$

The CIF for GFP$^+$ cell division given PDGF and FGF (CR category 1) in generation > 0 was modelled using the single factors PDGF and FGF, and the Fine and Gray link function. Simulated CIFs are shown in Figure 4f (green line) and results for parametric terms are shown in Supplementary Table S18.

The CIF for PDGFRα$^-$ (GFP$^-$) cell division (CR category 2) in generation > 0 was modelled using the single factor FGF (knowing that PDGF had not effect on GFP$^-$ cells) and the Fine and Grey link function. The model is shown in equation (S5.11).

Equation S5.11

$$\text{division CIF} \sim 1 + \text{const(FGF)}$$

Simulated CIFs derived from this model are shown in Fig. 4f (black line) and results for parametric terms are shown in Supplementary Table S18.

Supplementary Table S18 | Results from the semi-parametric model (shown in equation (S5.11)) describing the effect of cytokines on division for GFP+ and GFP- cells in generation > 0.

GFP$^+$					
**Parametric covariate**	**Coefficient**	**SE**	**Robust SE**	**z**	**P-value**
**const(FGF)**	0.779	0.223	0.223	3.5	0.00047
**const(PDGF)**	0.606	0.216	0.216	2.81	0.00497
**GFP$^-$**					
**const(FGF)**	-0.51	0.293	0.293	-1.74	0.0814

d) Generation of GFP$^+$ and GFP$^-$ daughters from GFP$^+$ and GFP$^-$ mothers

To study the renewal of GFP$^+$ and GFP$^-$ cells, mothers and daughters were gated for their GFP status at the end of their lifetime (i.e. before death, division, and right-censoring). Using these classifications, mother-daughter fate outcomes were divided into the following categories:

1) GFP$^+$ daughters derived from GFP$^+$ mothers
2) GFP$^-$ daughters derived from GFP$^+$ mothers
3) GFP$^+$ daughters derived from GFP$^-$ mothers
4) GFP$^-$ daughters derived from GFP$^-$ mothers

These CR categories were used to identify fate outcomes of interest in a CRR model that included terms for PDGF and FGF, shown in equation (S5.12).

Equation S5.12

$$\text{division CIF} \sim 1 + \text{const(PDGF)} + \text{const(FGF)}$$

The Fine and Gray link function was used in this model. FGF and PDGF promoted renewal of GFP$^+$ daughters from GFP$^+$ mothers (category 1). The simulated CIF for the generation of GFP$^+$ daughters from GFP$^+$ mothers is shown in Figure 4H (green lines). Results for parametric terms are shown in Supplementary Table S19.

Both PDGF and FGF did not increase the probability of generation of GFP$^-$ daughters from GFP$^+$ mothers (category 2). The simulated CIF for the generation of GFP$^-$ daughters from GFP$^+$ mothers is shown in Figure 4h (black lines). Results for parametric terms are shown in Supplementary Table S19.

bFGF and PDGF did not promote renewal of GFP$^-$ daughters from GFP$^-$ mothers (category 3). The simulated CIF for the generation of GFP$^-$ daughters from GFP$^+$ mothers is shown in Figure S7c (green lines). Results for parametric terms are shown in Supplementary Table S19. Both PDGF and FGF did not increase the probability of generation of GFP$^+$ daughters from GFP$^-$ mothers (category 4). The simulated CIFs for

the generation of GFP^+ and GFP^- daughters from GFP^- mothers are shown in Fig. S8c (black lines), and results for parametric terms are shown in Supplementary Table S19.

Supplementary Table S19 | Results from the semi-parametric model (shown in equation (S5.12)) describing the effect of cytokines on the generation of GFP^- and GFP^+ daughters from GFP^- and GFP^+ mothers

**Category 1 – GFP^+ daughters derived from GFP^+ mothers**					
**Parametric covariate**	**Coefficient**	**SE**	**Robust SE**	**z**	**P-value**
**const(PDGF)**	0.595	0.261	0.261	2.28	0.0229
**const(FGF)**	0.811	0.276	0.276	2.94	0.00326
**Category 2 – GFP^- daughters derived from GFP^+ mothers**					
**Parametric covariate**	**Coefficient**	**SE**	**Robust SE**	**z**	**P-value**
**const(PDGF)**	0.428	0.553	0.553	0.774	0.439
**const(FGF)**	-0.643	0.473	0.473	-1.36	0.174
**Category 3 - GFP^+ daughters derived from GFP^- mothers**					
**Parametric covariate**	**Coefficient**	**SE**	**Robust SE**	**z**	**P-value**
**const(PDGF)**	-0.422	0.39	0.39	-1.080	0.279
**const(FGF)**	-0.108	0.395	0.395	-0.273	0.785
**Category 4 - GFP^- daughters derived from GFP^- mothers**					
**Parametric covariate**	**Coefficient**	**SE**	**Robust SE**	**z**	**P-value**
**const(PDGF)**	0.339	0.482	0.482	0.703	0.482
**const(FGF)**	0.371	0.463	0.463	0.801	0.423

e) Concordance analysis of *Pdgfra*-GFP$^+$ siblings

To analyse concordance in the fate of GFP$^+$ siblings a clustering variable (SisterClusterID) was included in the non-parametric CRR model for PDGFRα$^+$ cell division which included the effects of FGF and PDGF (generation>0), as shown in equation (S5.13).

Equation S5.13

$$\text{division CIF} \sim 1 + \text{PDGF} + \text{FGF} + \text{cluster(SisterClusterID)}$$

This model passed tests for non-significant effects (Supplementary Table 20) and all terms were shown to be time invariant (Supplementary Table S21).

Supplementary Table 20 | Results from the Supremum-test of significance applied to the model shown in equation (S5.13). The results from this test were used to identify non -significant effects (p>0.05) of cytokines on cCFU-F division in generation > 0 cells.

	Supremum-test of significance	p-value H_0: B(t)=0
**(Intercept)**	3.93	0
**FGF**	4.76	0
**PDGF**	4.52	0

Supplementary Table S21 | Results from the Kolmogorov-Smirnov test for time invariance applied to the model in equation (S5.13). The results from this test show constant (p>0.05) and non-constant (p<0.05) effects of cytokines on cCFU-F division in generation>0 cells.

	Kolmogorov-Smirnov test	p-value H_0:constant effect
**(Intercept)**	0.166	0.076
**FGF**	0.134	0.414
**PDGF**	0.134	0.364

The cross-odds ratio (COR) was calculated using the *cor.cif* (contained within the *mets* R package) function using the symmetry condition (sym=1). The pseudo-code below shows the implementation of the *cor.cif* function in R.

cor.cif(cif = cif.model.division, data = tempdata, cause1 = 1, cause2 = 1, sym = 1)

where *cif* was the output from the CRR model above, *data* contained cell lifetimes for division, death, and right-censoring. cause1 and cause2 were the fate outcomes of interest for each sibling, respectively. For example, cause1=1 and cause2=1 allowed for the *cor.cif* function to compute the COR for GFP$^+$ divisions, as defined in the categories described in Text 5c.

Below is the output from the *cor.cif* function, which showed that GFP$^+$ siblings had significantly symmetric fates (COR>>1, p<<0.001).

Cross odds ratio dependence for competing risks

Effect of cause1=1 on cause2=1 under symmetry=1

	log-Coefficient	***SE***	***z***	***P-val***	***Cross odds ratio***	***SE***
Intercept	*2.37*	*0.126*	*18.8*	*0*	*10.7*	*1.35*

In order to visualise the symmetry in GFP$^+$ cell fates the probandwise concordance probability was calculated using the concordance function (contained within the *mets* R package). Conditional and unconditional probabilities are plotted in Figure 5e.

f) Flow cytometry analysis of MSC surface markers

Flow cytometry analysis of MSC surface markers was used to test for expression of canonical MSC markers. This is a routine quality control assay for MSC cultures, as described by the International Society for Cell Therapy (ISCT) MSCs. Table S22 shows that cCFU-F cultured in SFM expressed MSC surface markers indicative of a high quality MSC culture. Furthermore, cCFU-F did not lose expression of the stem cell marker SCA-1 nor gain expression of the endothelial marker CD31.

Supplementary Table S22 | FACS analysis of MSC surface markers expressed by cCFU-F cultures after four passages *in vitro.* The table shows no significant change in expression of SCA1 and CD31, as well as maintenance of canonical MSC markers

**Condition**	**Surface antigen**						
	**SCA-1**	**CD44**	**CD105**	**CD90.2**	**CD34**	**CD31**	**CD45**
**FGF+TGF+PDGF**	100%	100%	92.4%	18.4%	<1%	<1%	<1%

6. Permutation and randomisation tests for cCFU-F sibling cell fate outcomes

Permutation tests were used to quantify similarity in cCFU-F sibling cell cycle times. To test for similarity in sibling cell cycle times we calculated the mean difference in cycle time between sibling cell pairs. For each sibling pair the difference in cycle time was calculated, and then averaged. This average was compared to the mean difference in cycle time between randomly sampled cell pairs. 10,000 random permutations of the data set were generated using Monte Carlo simulation by randomly sampling cell pairs (sibling pair label shuffling, without replacement) from the pooled cell population. For each random permutation the mean difference in cycle time across all cell pairs was calculated. A two-tailed, unpaired Student's *t*-test ($\alpha=0.05$) was used to determine if the observed mean difference in sibling cycle times was significantly different to that of random permutations.

7. References

1 Tai, B.-C., Wee, J. & Machin, D. Analysis and design of randomised clinical trials involving competing risks endpoints. *Trials* **12**, 127-127, doi:10.1186/1745-6215-12-127 (2011).

2 Kent, D. M., Alsheikh-Ali, A. & Hayward, R. A. Competing risk and heterogeneity of treatment effect in clinical trials. *Trials* **9**, 30-30, doi:10.1186/1745-6215-9-30 (2008).

3 Berry, S. D., Ngo, L., Samelson, E. J. & Kiel, D. P. Competing Risk of Death: An Important Consideration in Studies of Older Adults. *J. Am. Geriatr Soc.* **58**, 783-787, doi:10.1111/j.1532-5415.2010.02767.x (2010).

4 Rauch, G. *et al.* Competing time-to-event endpoints in cardiology trials: A simulation study to illustrate the importance of an adequate statistical analysis. *Eur. J. Prev. Cardiol.* **21**, 74-80 (2014).

5 Schulgen, G. *et al.* Sample sizes for clinical trials with time-to-event endpoints and competing risks. *Contemp. Clin. Trials* **26**, 386-396, doi:http://dx.doi.org/10.1016/j.cct.2005.01.010 (2005).

6 Satagopan, J. M. *et al.* A note on competing risks in survival data analysis. *Br. J. Cancer* **91**, 1229-1235 (2004).

7 Scheike, T. H. & Sun, Y. Q. On cross-odds ratio for multivariate competing risks data. *Biostatistics* **13**, 680-694, doi:DOI 10.1093/biostatistics/kxs017 (2012).

8 Scheike, T. & Zhang, M.-J. Analyzing competing risks data using the {R}-timereg package. *J. Stat. Softw.* **38**, 1-15 (2011).

9 Scheike, T. & Martinussen, T. in *Statistics for biology and health* (Springer, New York, 2006).

10 Gray, R. J. A Class of K-Sample Tests for Comparing the Cumulative Incidence of a Competing Risk. *Ann. Stat.* **16**, 1141-1154, doi:DOI 10.1214/aos/1176350951 (1988).

11 Tsiatis, A. Nonidentifiability Aspect of Problem of Competing Risks. *Proc. Nat. Acad. Sci. USA* **72**, 20-22, doi:Doi 10.1073/Pnas.72.1.20 (1975).

12 Scheike, T. H. & Zhang, M. J. Flexible competing risks regression modeling and goodness-of-fit. *Lifetime Data Anal.* **14**, 464-483, doi:DOI 10.1007/s10985-008-9094-0 (2008).

13 Fine, J. P. & Gray, R. J. A proportional hazards model for the subdistribution of a competing risk. *J.Am. Stat. Ass.* **94**, 496-509, doi:Doi 10.2307/2670170 (1999).

14 Duffy, K. R. *et al.* Activation-Induced B Cell Fates Are Selected by Intracellular Stochastic Competition. *Science* **335**, 338-341, doi:DOI 10.1126/science.1213230 (2012).

Supplementary Figures

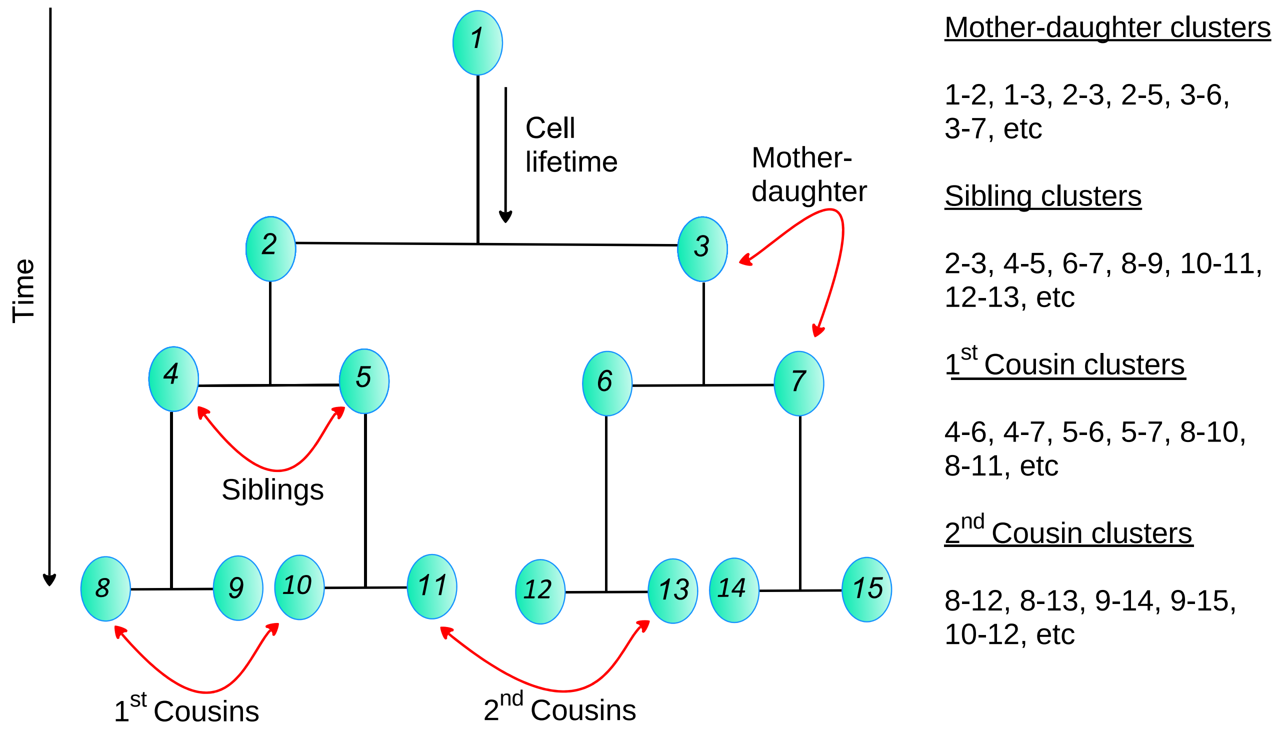

Supplementary Figure S1 | Single cell pedigrees showing kinship clustering. Kinship clusters are pairs of related cells within a pedigree including mother-daughter clusters, sibling clusters, 1st cousin clusters, and 2nd cousin clusters. Kinship clusters at different depths within a single cell pedigree have different degrees of familial and temporal separation.

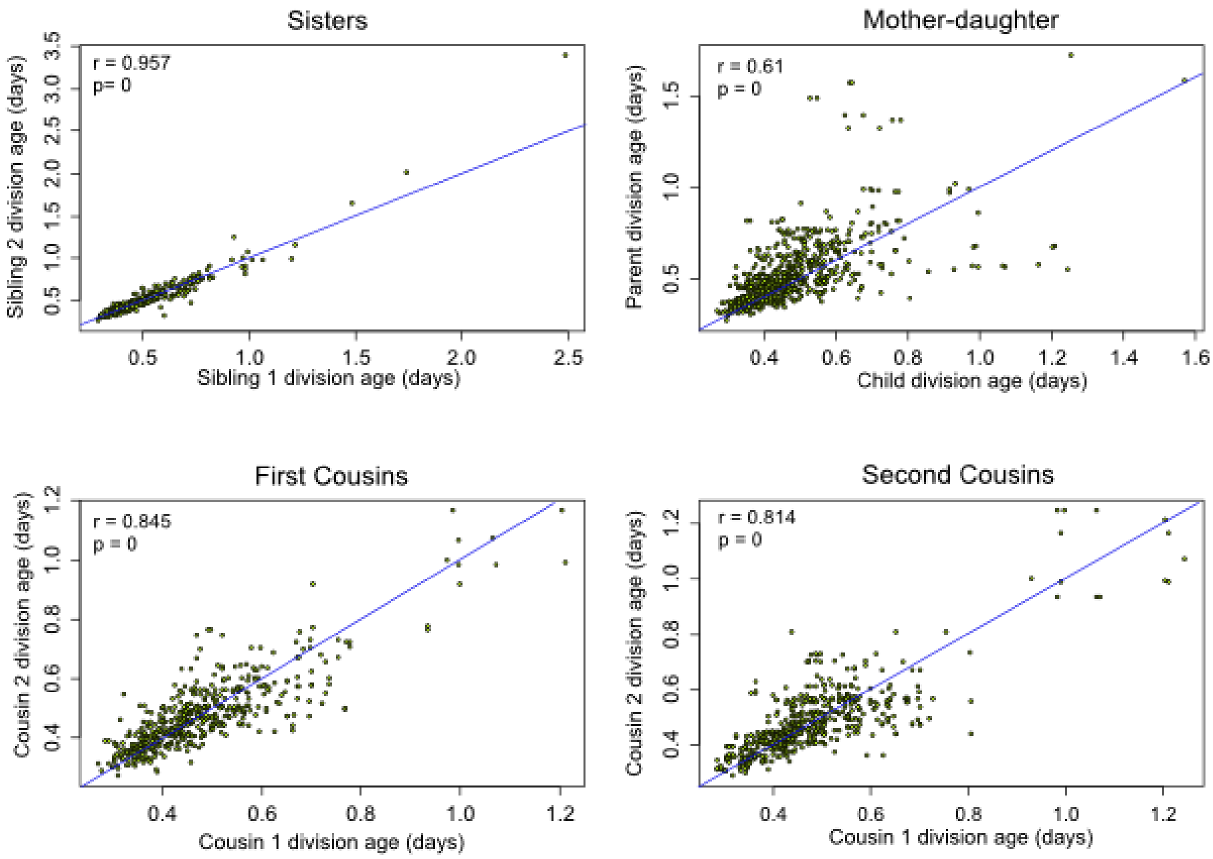

Supplementary Figure S2 | Bivariate plots showing correlation in division age between kinship relations (siblings, mother-daughters, 1st cousins, and 2nd cousins) for GFP-(GMP) cells. r is the Pearson's correlation coefficient.

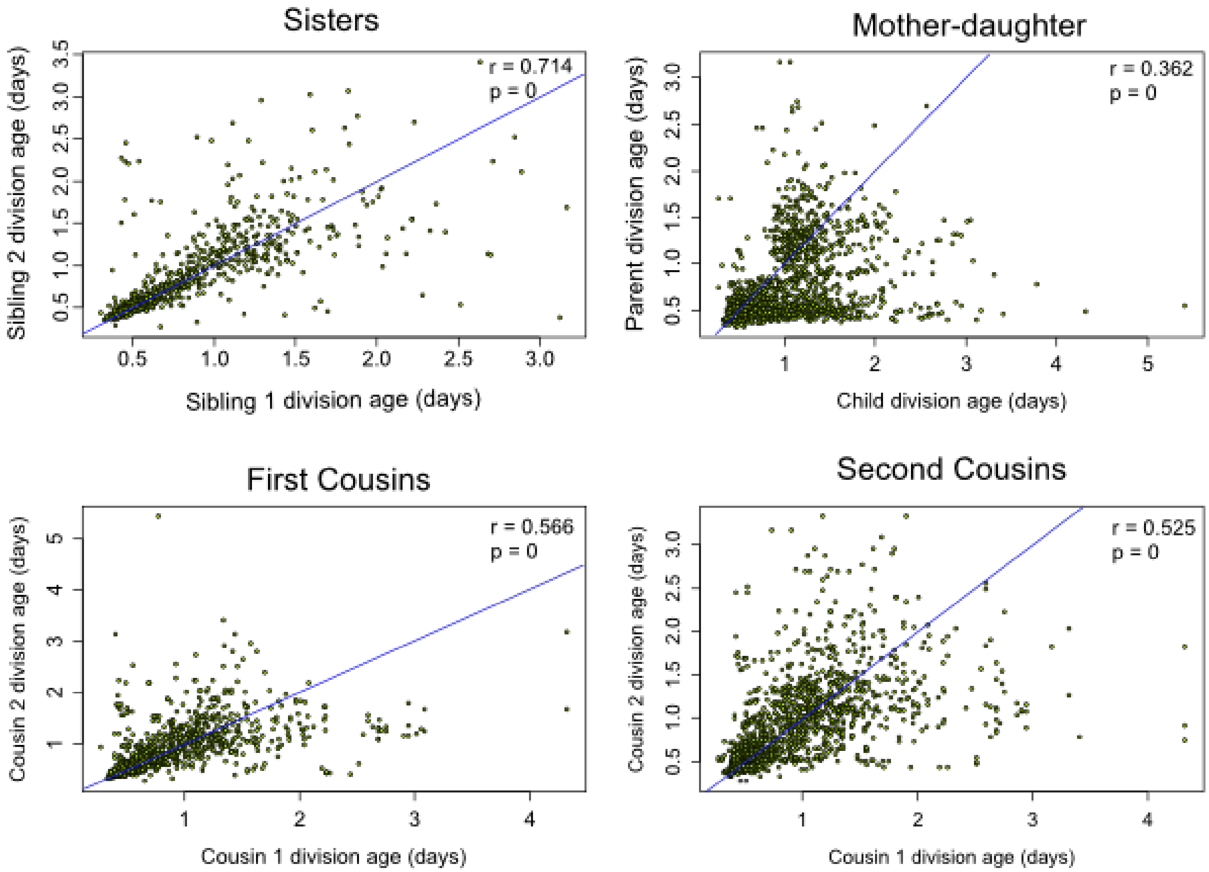

Supplementary Figure S3 | Bivariate plots showing the correlation in division age between kinship relations (siblings, mother-daughters, 1st cousins, and 2nd cousins) for GFP^{+} (differentiated) cells. r is the Pearson's correlation coefficient.

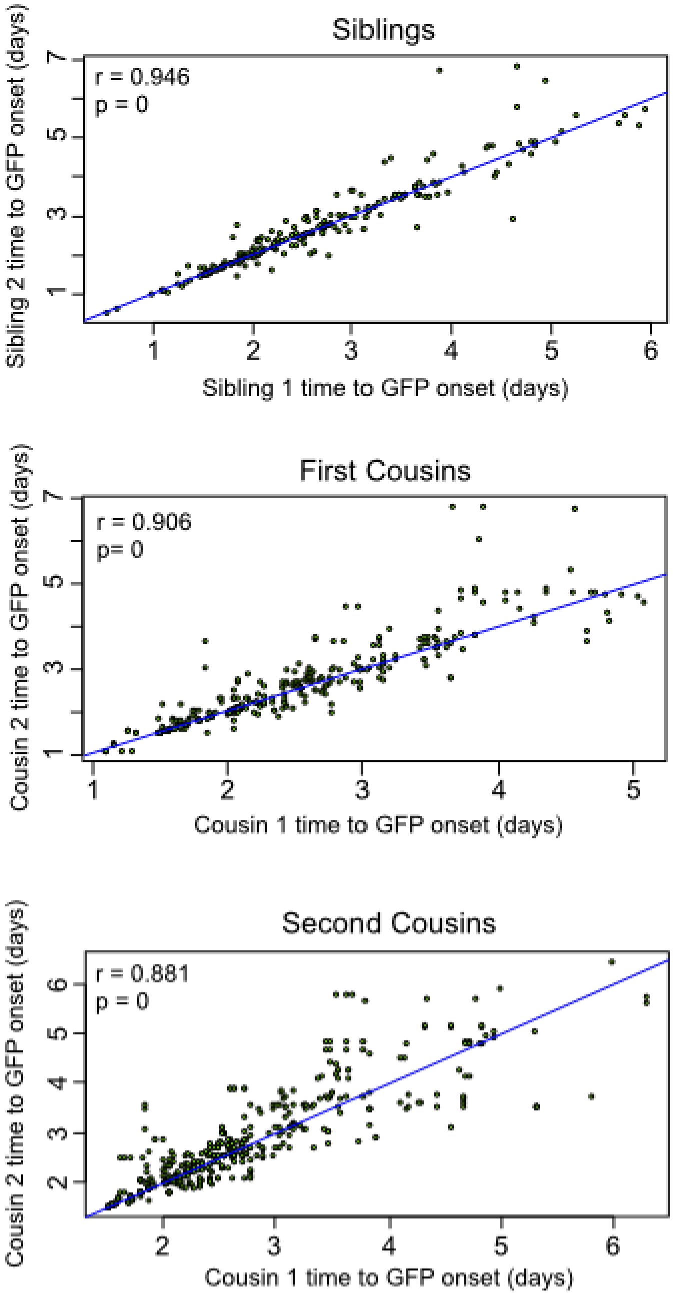

Supplementary Figure S4 | Bivariate plots showing correlation between kinship relations (siblings,1st cousins, and 2nd cousins) for the time elapsed between exposure to growth factor and GFP expression. r is the Pearson's correlation coefficient.

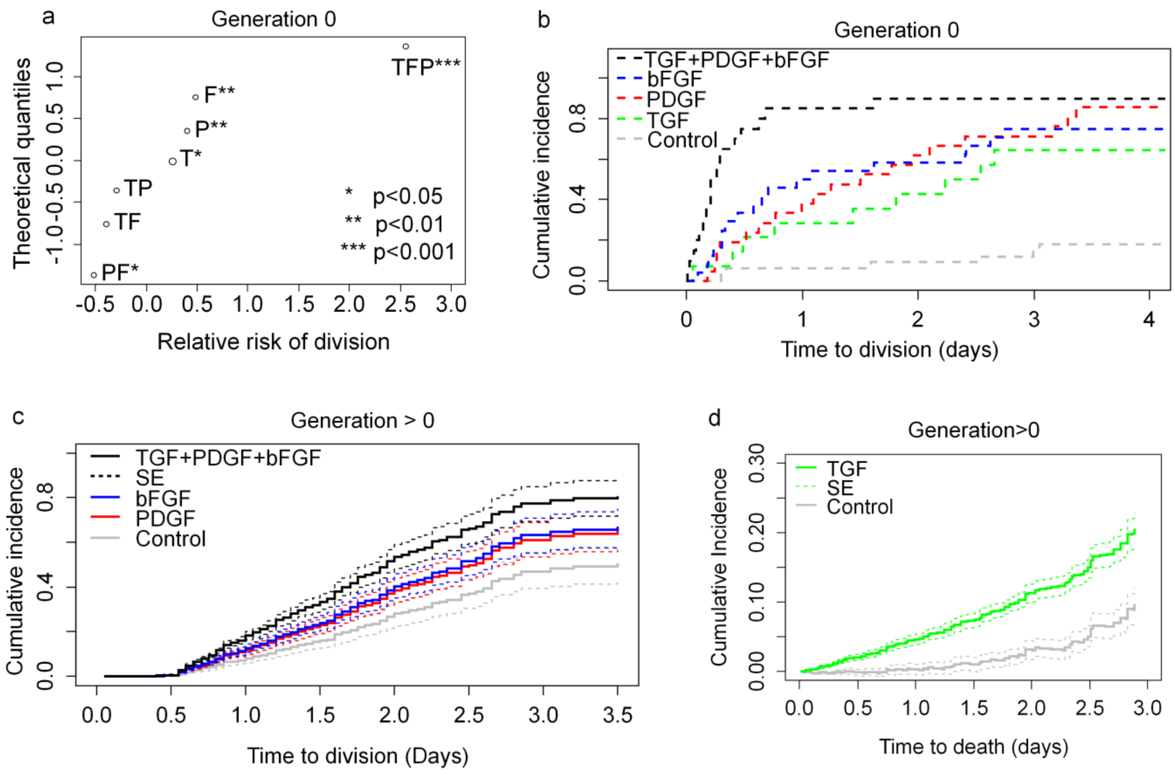

Supplementary Figure S5 | PDGF, FGF, and TGF affect division and death probabilities of cCFU-F in generation 0 and generation > 0 (cell birth is observed). A) Quantile plot showing relative risks of division (generation 0) for cytokines and their interactions. B) Empirical subdistributions (see *cuminc* in *cmprsk* package) illustrating the effect of TGF+PDGF+FGF (black) , FGF (blue), PDGF (red), TGF (green), and control (grey) on division probability for cCFU-F from generation 0 (cell birth not observed). C) CRR model illustrating the effect of PDGF:FGF:TGF (black) , FGF (blue), PDGF (red), and control (grey) on division probability for cells from generations > 0 (cell birth is observed). Note that cells will not divide for at least half a day after their birth. D) CRR model showing the effect of TGF alone on the death probability of cells in generation > 0. TGF alone (green) significantly increased the probability of death relative to control (grey).

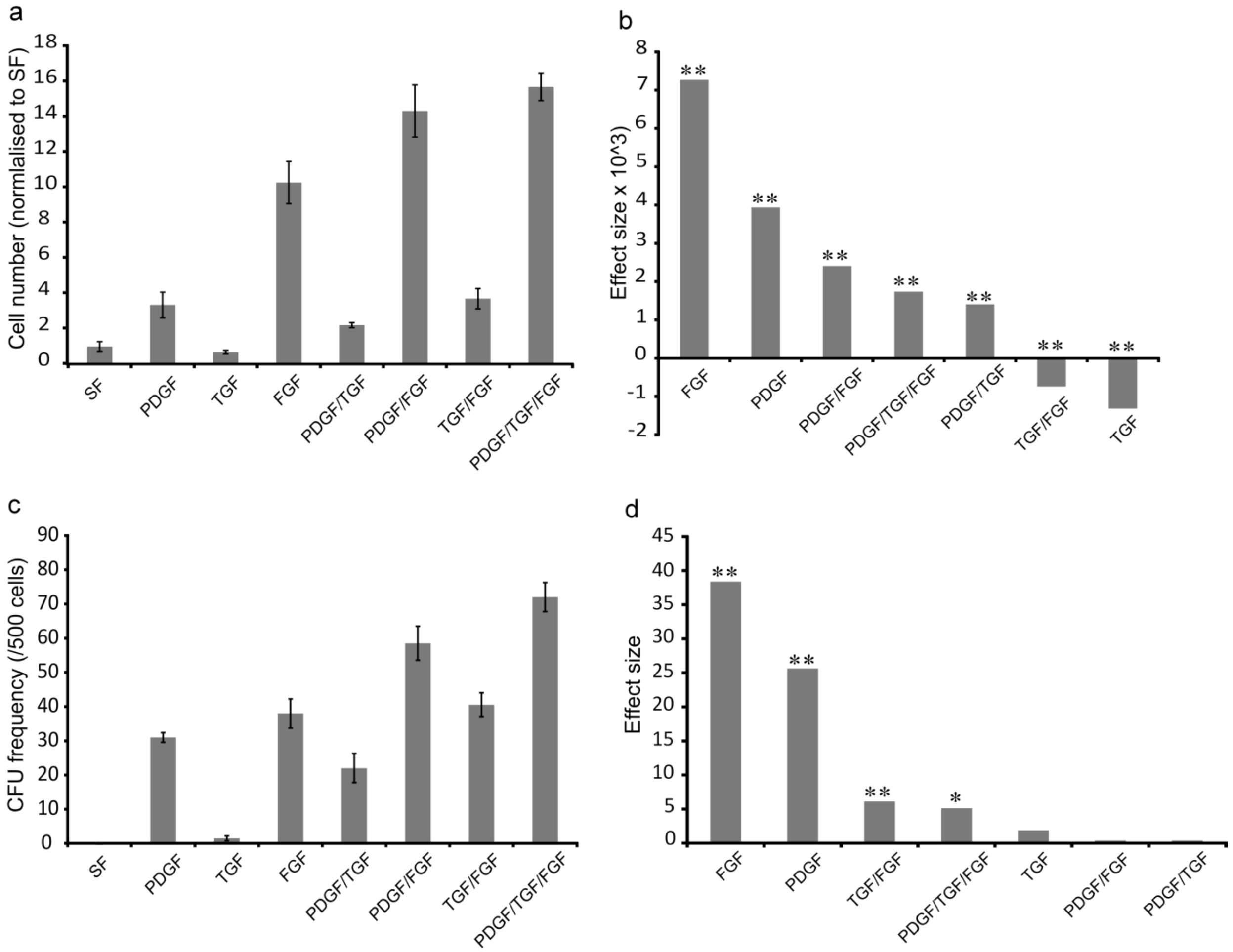

Supplementary Figure S6 | Cytokines stimulate cCFU-F proliferation and colony formation in response to cytokine combinations. A) Cell numbers obtained from culturing cCFU-F in SFM in all combinations PDGF, TGF, and FGF. B) Single factor and interaction effects of FGF, TGF, PDGF in SFM with no cytokines. Effects are calculated from contrasts (*SI Appendix*, Text S3A). Significant single factor effects included FGF (p = 1.4E-08) and PDGF (p = 1.7E-06). Significant interactions included PDGF/FGF (p = 6.6E-05), PDGF/FGF/TGF (p = 6.1E-04). C) CFU frequency in various combinations of cytokines and with no cytokines (SF). D) Calculated effect sizes for cytokines on CFU-F colony formation. Main and interaction effects of FGF, TGF, PDGF in serum free medium with no cytokines. Significant effects included FGF (p = 1.64E-08), PDGF (p = 3.87-07), TGF/FGF (p = 0.007), and PDGF/TGF/FGF (p = 0.011). Y-axes show effect size in arbitrary units.

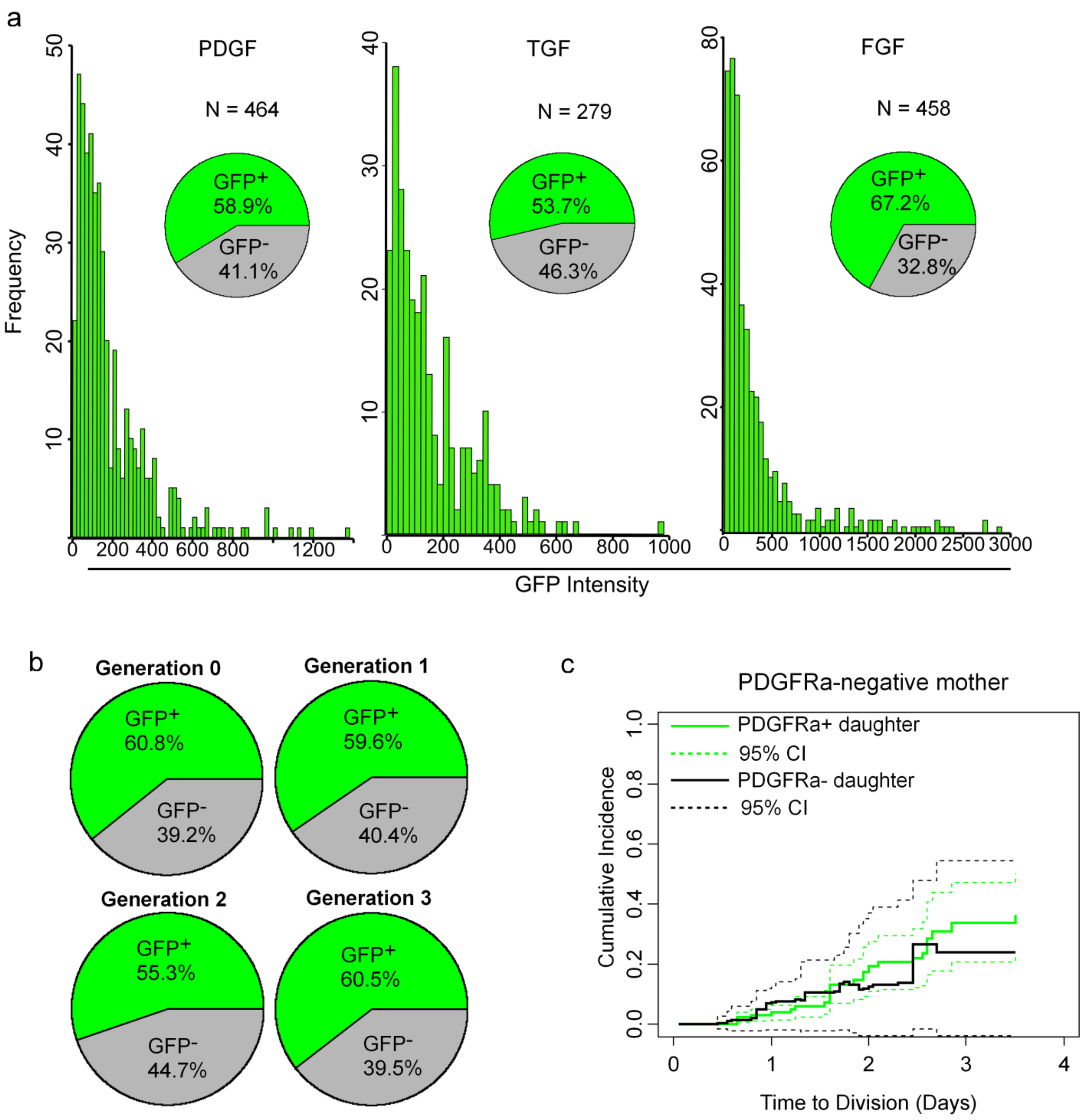

Supplementary Figure S7 | *Pdgfra*-GFP expression varied widely within each condition and also uniquely between each condition. A) Histograms showing cCFU-F *Pdgfra*-GFP expression during passage in SFM containing PDGF, TGF, and FGF. Pie-charts show the percentage of positive and negative cells after thresholding (threshold of 100 fluorescent units). B) GFP^- cells give rise to both GFP^+ and GFP^- cells at a low frequency and in equal proportions. C) CRR model showing the probability of division for GFP^+ and GFP^- daughters derived from GFP^- mothers.
